# PD-L1 transmembrane domain substitution overcomes CAR-T exhaustion driven by tonic signaling

**DOI:** 10.1016/j.omton.2026.201364

**Published:** 2026-10-14

**Authors:** Liang Zhang, Xiang Xu, Haiwen Yu, Shizhen Qiu, Zhitong Wang, Yinying Chu, Guangfei Li, Yi Fang, Peijie He, Haitao Wu, Lei Cheng, Hongxiu Yu, Haopeng Wang, Jian Chen

**Affiliations:** 1 ENT Institute and Department of Otorhinolaryngology, Eye & ENT Hospital, Fudan University, Shanghai 200031, China; 2 Shanghai Key Laboratory of Gene Editing and Cell Therapy for Rare Diseases, Fudan University, Shanghai 200031, China; 3 School of Life Science and Technology, ShanghaiTech University, Shanghai 201210, China; 4 Institute of Biomedicine Sciences & Shanghai Stomatological Hospital, Fudan University, Shanghai, China; 5 Changping Laboratory, Beijing 102206, China

**Keywords:** CAR-T, transmembrane domain, tonic signaling, T cell exhaustion, PD-L1, GD2, CSPG4

## Abstract

Chimeric antigen receptor (CAR)-T cell therapy has achieved limited success against solid tumors, in part because excessive tonic signaling drives premature T cell exhaustion, as exemplified by CSPG4 and GD2 CARs. Here, we demonstrate that replacing the dimeric CD28 transmembrane (TM) domain with the monomeric PD-L1 TM domain substantially mitigates tonic signaling. The PD-L1 TM displays a smaller tilt angle and lower hydrophobic thickness, enabling a better membrane fit that minimizes spontaneous CAR clustering. This structural feature reduces tonic signaling and exhaustion and confers superior proliferation and memory preservation, resulting in enhanced and sustained tumor killing *in vitro* and *in vivo*. Mechanistically, these benefits were associated with attenuated phosphorylation across the CD3ζ-ZAP70-PLC-γ1/MAPK/AKT/NF-κB axis, downregulation of exhaustion-associated genes, and upregulation of stemness- and memory-related programs. Collectively, our findings establish that the PD-L1 TM swap is a modular and broadly applicable strategy to suppress CAR tonic signaling and promote durable, memory-biased CAR-T responses against solid tumors.

## Graphical abstract

**Figure:**
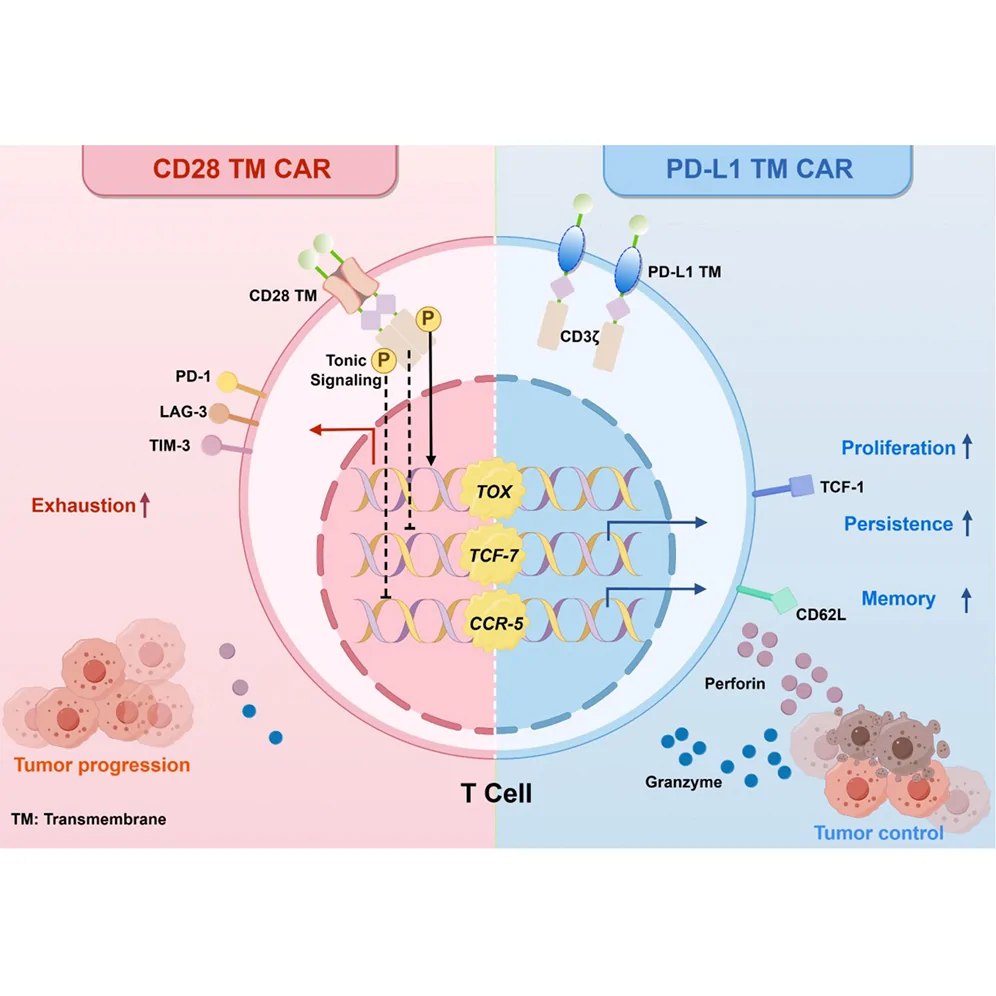

## Teaser

CAR-T therapy against solid tumors is limited by tonic signaling-driven exhaustion. Replacing the CD28 transmembrane domain with the PD-L1 transmembrane domain reduces CAR tonic signaling and exhaustion by improving membrane fit and minimizing receptor clustering, offering a broadly applicable approach to improve CAR-T efficacy.

## Introduction

Chimeric antigen receptor (CAR) T cell therapy has achieved unprecedented success in treating hematological cancers,^1^^,^^2^^,^^3^ but outcomes in solid tumors remain challenging.^4^^,^^5^^,^^6^ Solid malignancies present unique and formidable obstacles to adoptive T cell therapy, including a profoundly immunosuppressive tumor microenvironment, heterogeneous target antigen expression, and intrinsic T cell dysfunction.^7^^,^^8^ Well-characterized solid tumor antigens such as CSPG4, GD2, and mesothelin are frequently targeted by CAR-T cells.^9^^,^^10^^,^^11^ However, the corresponding CARs often exhibit high levels of antigen-independent tonic signaling, a major driver of rapid T cell dysfunction and exhaustion.^12^^,^^13^

To mitigate tonic signaling, various strategies have been explored. Engineering alternative costimulatory domains is one approach: substituting 4-1BB for CD28 costimulation can reduce T cell exhaustion by promoting slower differentiation rates.^14^^,^^15^ Other methods include adjusting scFv affinity or CAR expression level to avoid overactivation,^16^^,^^17^ as well as modifying hinge/spacer lengths to tune synapse formation.^18^^,^^19^ More sophisticated solutions involve elaborate intracellular circuits or gene edits, such as CRISPR knockout of inhibitory pathways or artificial intelligence (AI)-driven design of CAR constructs.^20^^,^^21^^,^^22^^,^^23^ While these interventions can enhance CAR-T function, they are often complex, time-consuming, or antigen specific.^20^^,^^22^ Therefore, a simple and broadly applicable design strategy that directly targets and reduces tonic signaling remains an unmet need.

Given its role in receptor assembly,^24^ we investigated the CAR transmembrane (TM) domain as a potential regulator of tonic signaling. The commonly used CD28 TM contains glycine-zipper motifs that drive dimerization.^25^^,^^26^ Such intrinsic dimerization may promote spontaneous CAR clustering on the T cell surface. In contrast, TM domains from other immune receptors can adopt distinct oligomerization states, including monomeric forms.^27^^,^^28^^,^^29^^,^^30^ This led us to hypothesize that substituting the dimeric CD28 TM with a naturally monomeric domain would reduce undesired self-association and lower tonic signaling.

To this end, we generated a series of CARs with the CD28 TM replaced by monomeric TMs from CD86, PDGFRB, or PD-L1 (Figure 1A).^28^^,^^29^^,^^30^ We systematically evaluated these constructs in Jurkat cells and primary human T cells, measuring CAR expression, membrane distribution, tonic signaling, and antigen-specific responses. Strikingly, across CARs of different specificities, the PD-L1 TM consistently yielded the most favorable functional profile, achieving minimal tonic signaling without compromising antigen-driven responses. Collectively, our findings establish the PD-L1 TM swap as a straightforward and broadly applicable strategy to optimize CAR-T cell fitness for solid tumor therapy.

**Figure 1:**
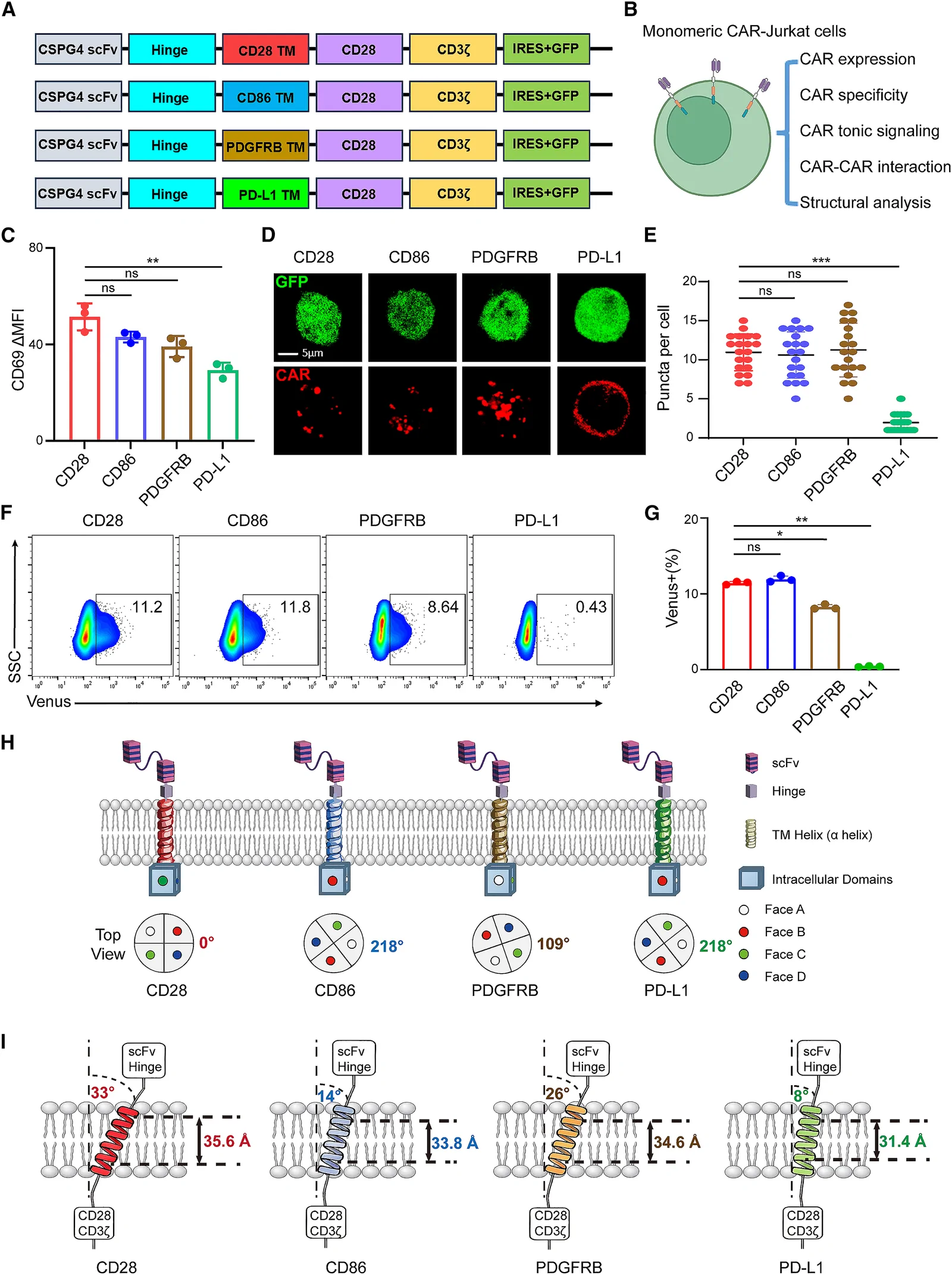
PD-L1 transmembrane domain reduces CAR tonic signaling by attenuating spontaneous CAR clustering in Jurkat cells (A) Schematic representation of CSPG4-targeted CAR constructs. (B) Schematic overview of the experimental workflow. (C) Quantification of tonic signaling, measured as CD69 mean fluorescence intensity (MFI) in CAR-transduced Jurkat cells cultured in the absence of antigen. Data represent the mean ± SEM of *n* = 3 biologically independent experiments. *p* values were determined by unpaired Student’s *t* test relative to CD28 TM. ∗∗*p* < 0.01; n.s., not significant. (D) Representative immunofluorescence images showing CAR distribution on the surface of fixed Jurkat cells. CAR was stained with an anti-hIgG antibody and imaged by confocal microscopy. Scale bar, 5 μm. (E) Quantification of spontaneous CAR clustering. The number of distinct CAR clusters per cell was counted from immunofluorescence images. Data represent the mean ± SEM of 20 randomly selected cells analyzed per group. ∗∗∗*p* < 0.001; n.s., not significant; unpaired Student’s *t* test relative to CD28 TM. (F) Representative flow cytometry plots from a bimolecular fluorescence complementation (BiFC) assay in live Jurkat cells. CAR constructs were fused to N- and C-terminal fragments of Venus fluorescent protein, and CAR-CAR interaction reconstitutes Venus fluorescence. Numbers indicate the percentage of Venus^+^ cells within the CAR^+^ population. (G) Quantification of BiFC Venus^+^ cell frequencies from independent experiments. Data represent the mean ± SEM of *n* = 3 biologically independent experiments. ∗*p* < 0.05 and ∗∗*p* < 0.01; n.s., not significant; unpaired Student’s *t* test relative to CD28 TM. (H) Predicted torsional reorientation angles of the TM helices for each CAR construct, derived from atomistic molecular dynamics simulations. (I) Predicted tilt angles (left) and hydrophobic thicknesses (right) of the TM domains for each CAR construct, derived from structural modeling.

## Results

### A monomeric PD-L1 TM domain reduces tonic signaling and spontaneous CAR clustering

We previously identified CSPG4 as a therapeutic target in head and neck squamous cell carcinoma, motivating our focus on CSPG4 CAR-T cells.^31^ However, their efficacy is constrained by excessive tonic signaling.^10^ To test whether replacing the dimeric CD28 TM domain with a naturally monomeric TM domain could reduce antigen-independent tonic signaling, we constructed a series of CSPG4-specific CARs. These CARs shared the same CSPG4 scFv, CD28 costimulatory domain, and CD3ζ chain, but the CD28 TM was substituted with the TM domains from CD86, PDGFRB, or PD-L1, which have been reported to adopt a monomeric conformation in their native contexts (Figure 1A). The resulting constructs were expressed in Jurkat cells to evaluate CAR surface expression, antigen specificity, tonic signaling, intermolecular proximity, and structural properties (Figure 1B). All four CAR constructs were expressed at comparable levels on Jurkat cells and retained the ability to recognize CSPG4, as shown by CD69 upregulation upon co-culture with K562-CSPG4 cells (Figures S1A–S1D).

As a readout for CAR tonic signaling, we next measured CD69 expression, an early T cell activation marker previously established as an indicator of pre-T cell receptor (TCR) and pre-B cell receptor (BCR) tonic signaling strength.^32^^,^^33^ CD28 TM CAR-Jurkat cells exhibited the highest basal CD69 levels. CD86 and PDGFRB TM CARs showed modest reductions that did not reach statistical significance, whereas the PD-L1 TM CAR displayed the lowest CD69 expression (Figures 1C and S1E). These data indicate that among the monomeric TM variants tested, only the PD-L1 TM significantly suppresses tonic signaling in Jurkat cells. To visualize CAR distribution on the cell surface, we performed immunofluorescence staining. CARs bearing CD28, CD86, or PDGFRB TM domains formed prominent punctate clusters, whereas the PD-L1 TM CAR exhibited a diffuse and uniform membrane distribution (Figure 1D). Quantification confirmed a significant reduction in clustering exclusively for the PD-L1 TM CAR (*p* < 0.001; Figure 1E). To rule out potential fixation artifacts, we used a bimolecular fluorescence complementation (BiFC) assay, in which split Venus fragments fused to the CAR intracellular tail report CAR-CAR proximity in live cells. Robust Venus fluorescence was observed in CD28, CD86, and PDGFRB TM CARs but was essentially absent in the PD-L1 TM CAR (Figures 1F, 1G, and S1F). Thus, the PD-L1 TM drives a predominantly monomeric CAR state, whereas CD86 and PDGFRB TMs still permit substantial self-association in this context.

To explore the structural basis of this difference, we compared the predicted structures of the four CAR ectodomain-TM-juxtamembrane segments (Figure S1G). Although CD86 and PD-L1 TMs were slightly shorter than CD28 and PDGFRB TMs, their clustering and tonic signaling phenotypes diverged markedly, suggesting that TM length is not a primary determinant of CAR self-association. We next considered torsional reorientation of the intracellular signaling domains, as alanine insertions that alter the rotation of the CAR TM helix have been shown to modulate *in vivo* antitumor efficacy, with a 2-alanine insertion (218° rotation) producing the optimal functional outcome.^34^ Analysis of our constructs revealed that the PD-L1 TM also produced a 218° rotation relative to the CD28 TM, a feature it shared with the CD86 TM (Figure 1H). Although the CD86 TM matched this optimal torsion, it still exhibited strong clustering and tonic signaling, suggesting that torsional alignment alone is insufficient to explain the monomeric behavior of the PD-L1 TM CAR.

Because the CARs differed only in their TM domains, we hypothesized that TM-dependent biophysical interactions with the lipid bilayer, such as hydrophobic mismatch, might govern self-association.^35^^,^^36^ We therefore performed PPM (positioning of proteins in membranes) analysis, which estimates the orientation and energetics of TM helices within a lipid bilayer. The PD-L1 TM displayed the smallest tilt angle (8° ± 2°) and the lowest hydrophobic thickness (31.4 ± 1.7 Å) among the four TMs, whereas CD28 (33° ± 1°, 35.6 ± 1.8 Å), CD86 (14° ± 4°, 33.8 ± 2.1 Å), and PDGFRB (26° ± 3°, 34.6 ± 1.9 Å) exhibited greater membrane mismatch (Figure 1I).^37^ These features suggest that the PD-L1 TM imposes less hydrophobic insertion pressure and has a reduced thermodynamic drive to self-associate, providing a plausible structural explanation for its monomer-enriched state.

Similarly, CAR-T cells targeting GD2 and mesothelin, two additional solid tumor antigens of clinical interest, frequently exhibit high tonic signaling that limits their therapeutic potential.^9^^,^^11^ We next tested the generalizability of this strategy in GD2- and mesothelin-targeted CARs. In GD2 CARs, only the PD-L1 TM significantly reduced tonic CD69 expression, whereas CD86 and PDGFRB TMs did not, and antigen-induced activation was preserved (Figures S2A–S2D). Mesothelin CARs showed the same pattern (Figures S2E–S2H). Thus, the PD-L1 TM swap consistently attenuates tonic signaling across multiple CAR specificities without impairing antigen responsiveness. Given this unique advantage of the PD-L1 TM over the other monomeric TMs, we focused our subsequent studies on characterizing the optimized functional phenotype of PD-L1 TM CAR-T cells.

### The PD-L1 TM attenuates tonic signaling-driven exhaustion and preserves a less differentiated phenotype in primary human CAR-T cells

To determine whether the PD-L1 TM maintains its favorable properties in primary human T cells, we transduced activated T cells with CARs containing CD28 or PD-L1 TM domains and monitored tonic signaling, exhaustion, proliferation, and memory differentiation (Figure 2A). A bead-activated, untransduced T cell group (Beads) served as a control to distinguish CAR-intrinsic tonic signaling from background activation. Both CAR constructs were expressed at comparable levels on the T cell surface (Figure 2B). We tracked activation markers from day 7 through day 19, capturing the entire period until the Beads group ceased expanding, which also encompasses the 7- to 14-day window widely used in the CAR-T field to evaluate tonic signaling-driven exhaustion.^15^^,^^38^^,^^39^^,^^40^^,^^41^ CD28 TM CAR-T cells consistently displayed significantly elevated 4-1BB, CD69, CD25, and ICOS levels relative to the Beads control, and this difference represents the magnitude of CAR tonic signaling. PD-L1 TM substitution markedly reduced all these markers. Notably, CD69, CD25, and ICOS levels in PD-L1 TM CAR-T cells approached those of the Beads control, indicating that this modification effectively suppresses tonic signaling (Figures 2C–2F). A similar pattern was observed for inhibitory receptors. PD-1, Lag-3, and Tim-3 levels were substantially elevated in CD28 TM CAR-T cells compared with the Beads group, whereas PD-L1 TM CAR-T cells showed significant reductions across all three markers (Figures 2G–2I). The maximal differences between CD28 and PD-L1 TM groups occurred around day 10–13 for both activation and exhaustion markers.

**Figure 2:**
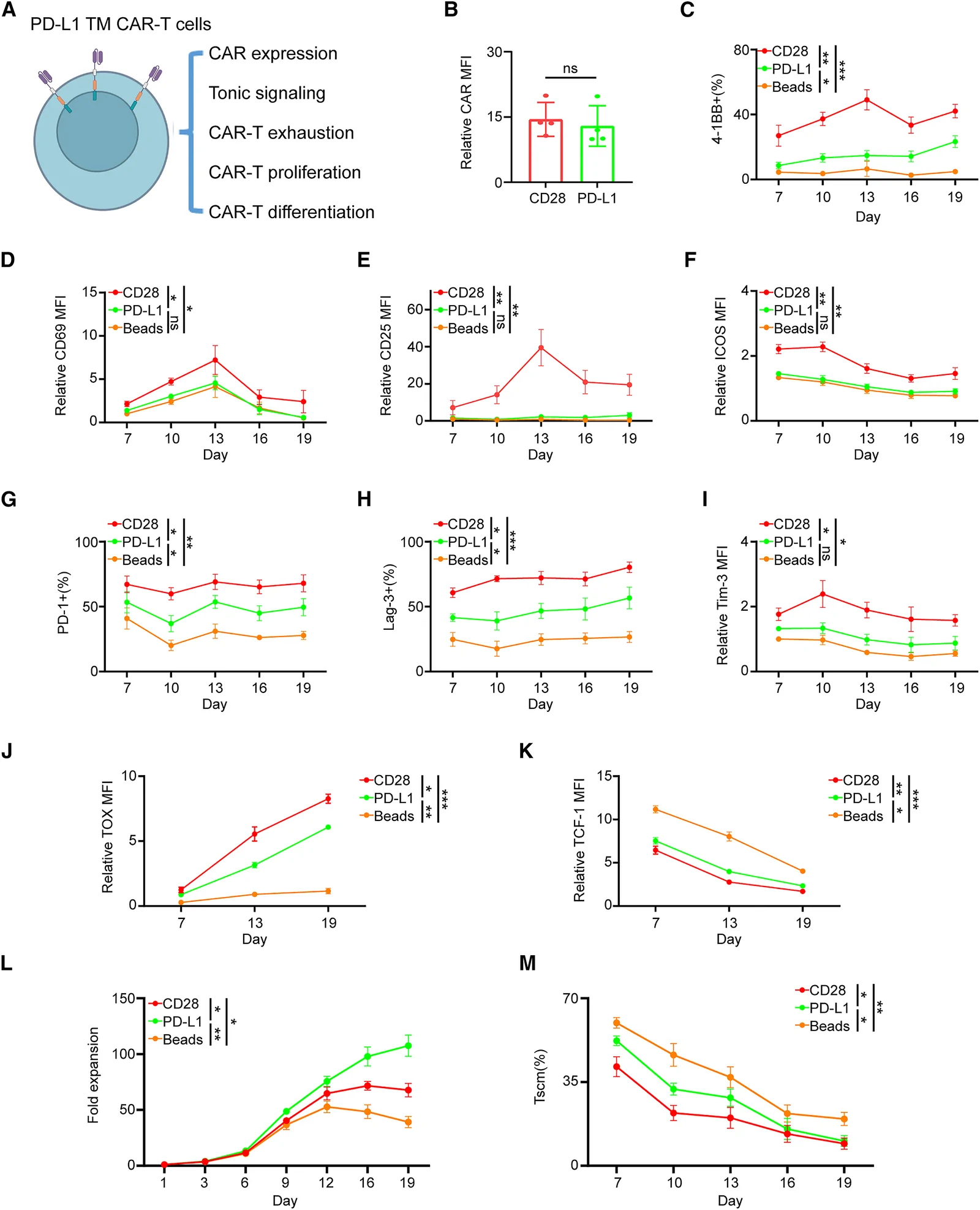
PD-L1 TM attenuates tonic signaling-driven exhaustion and preserves a less differentiated phenotype in primary human CAR-T cells (A) Schematic of the experimental workflow for evaluating PD-L1 TM CAR-T cell phenotypes in primary human T cells. Human T lymphocytes were activated with CD3/CD28 Dynabeads and transduced 48 h later with lentivirus expressing the indicated second-generation CSPG4 CAR. CAR surface expression was assessed by anti-human IgG (hIgG) staining on day 14 post-transduction. Time points for all other assays are indicated in the respective figures; cells were maintained in culture for up to 20 days until experiments were completed. (B) CAR surface expression detected by anti-hIgG antibody staining and presented as relative MFI normalized to isotype control. Data represent the mean ± SEM of *n* = 4 biologically independent experiments, each performed with a different donor. (C–F) Longitudinal quantification of the activation markers 4-1BB (C), CD69 (D), CD25 (E), and ICOS (F) from day 7 to day 19. CD69, CD25, and ICOS values were normalized to the day 7 Beads control within each experiment and are presented as fold change relative to baseline. Data represent the mean ± SEM of *n* = 3 biologically independent experiments, each performed with a different donor. (G–I) Longitudinal quantification of the inhibitory receptors PD-1 (G), Lag-3 (H), and Tim-3 (I) from day 7 to day 19. Data represent the mean ± SEM of *n* = 3 biologically independent experiments, each performed with a different donor. (J and K) Longitudinal quantification of TOX (J) and TCF-1 (K) relative MFI from day 7 to day 19. Data represent the mean ± SEM of *n* = 3 biologically independent experiments, each performed with a different donor. (L) Cumulative fold expansion over 19 days. Data represent the mean ± SEM of *n* = 3 biologically independent experiments, each performed with a different donor. (M) Tscm (CD45RA^+^CD62L^+^) frequency from day 7 to day 19. Memory subsets were defined as Tscm (CD45RA^+^CD62L^+^), Tcm (CD45RA^−^CD62L^+^), Tem (CD45RA^−^CD62L^−^), and Teff (CD45RA^+^CD62L^−^). Tscm, stem cell memory T cells; Tcm, central memory T cells; Tem, effector memory T cells; Teff, effector T cells. Data represent the mean ± SEM of *n* = 3 biologically independent experiments, each performed with a different donor.

Because inhibitory receptors can also be upregulated as part of normal T cell activation, we next examined TOX, a master transcription factor that drives terminal exhaustion and more specifically reflects the exhaustion commitment. TOX expression remained near baseline in the Beads control but rose progressively in both CAR groups, and this increase was significantly attenuated in PD-L1 TM CAR-T cells relative to CD28 TM CAR-T cells (Figure 2J). We further assessed TCF-1, a transcription factor essential for stemness and memory maintenance that also identifies an early progenitor exhausted subset. TCF-1 levels declined in all groups over time but were best preserved in the Beads group, followed by PD-L1 TM CAR-T cells, and were most severely reduced in CD28 TM CAR-T cells (Figure 2K). Together, these data indicate that the elevated inhibitory receptor expression in CD28 TM CAR-T cells reflects genuine exhaustion commitment rather than transient activation and that the PD-L1 TM substantially mitigates this exhaustion program.

Consistent with reduced tonic signaling, PD-L1 TM CAR-T cells displayed the most durable proliferation, continuing to expand through day 19 without a plateau, whereas CD28 TM CAR-T cells peaked at day 16 and the Beads control at day 12 (Figure 2L). A corresponding hierarchy was observed in memory preservation, as assessed by CD62L and CD45RA staining, a standard approach for distinguishing memory subsets in CAR-T cells.^42^^,^^43^^,^^44^ The highest stem cell memory T cells (Tscm) frequencies were found in the Beads control, intermediate levels in PD-L1 TM CAR-T cells, and the lowest in CD28 TM CAR-T cells (Figure 2M), a pattern that extended across the entire Tscm and central memory T cells (Tcm) compartment (Figure S3A).

We next compared the PD-L1 TM CSPG4 CAR with our previously reported scFv-optimized mutant M5, which carries reduced positive charge patches to lower intrinsic tonic signaling (Figure S3B). All three constructs were expressed at comparable levels (Figure S3C). M5 CAR-T cells showed reduced tonic signaling relative to the parental CD28 TM CAR, although their tonic markers remained slightly higher than those of PD-L1 TM CAR-T cells, whereas exhaustion markers and memory preservation were comparable between the two optimized constructs (Figures S3D and S3E). In proliferation assays, M5 CAR-T cells outperformed the CD28 TM CAR but remained modestly below PD-L1 TM CAR-T cells (Figure S3F). These data show that the PD-L1 TM swap achieves functional improvements approaching those of scFv optimization, despite being a structurally simpler and antigen-independent modification.

We next examined whether the PD-L1 TM conferred similar benefits in GD2-directed CAR-T cells. Both CAR constructs were expressed at comparable levels on primary T cells (Figure S4A). PD-L1 TM GD2 CAR-T cells showed markedly reduced spontaneous clustering (Figures S4B and S4C), lower expression of tonic signaling markers and phospho-ZAP70 (Figures S4D–S4G), and a significant decrease in exhaustion markers (Figures S4H–S4J). Functionally, they exhibited enhanced expansion and retained a higher proportion of Tscm cells (Figures S4K and S4L). These data establish that the PD-L1 TM swap is a broadly applicable strategy to attenuate tonic signaling-driven exhaustion and preserve memory differentiation across multiple CAR specificities.

### PD-L1 TM CAR-T cells exhibit superior and sustained tumor cell killing *in vitro*

To assess whether the reduced tonic signaling and preserved memory phenotype of PD-L1 TM CAR-T cells translate into enhanced antitumor function, we performed a series of *in vitro* assays evaluating cytotoxicity, antigen-induced differentiation, cytokine secretion, activation dynamics, and functional persistence under repetitive antigen challenge (Figure 3A). We first confirmed CSPG4 and GD2 expression on the respective target cells (Figures S5A and S5B). Using a luciferase-based cytotoxicity assay,^45^ PD-L1 TM CSPG4 CAR-T cells displayed significantly stronger killing than their CD28 TM counterparts (Figure 3B), a result that was recapitulated in GD2 CAR-T cells against Nalm6-GD2 targets (Figure S5C). We also compared PD-L1 TM CAR-T cells with our previously reported scFv-optimized CSPG4 CAR (M5). The two constructs exhibited comparably enhanced cytotoxicity relative to the parental CD28 TM CAR (Figure S5D), demonstrating that TM engineering can achieve functional gains approaching those of scFv optimization through a simpler, modular design.

**Figure 3:**
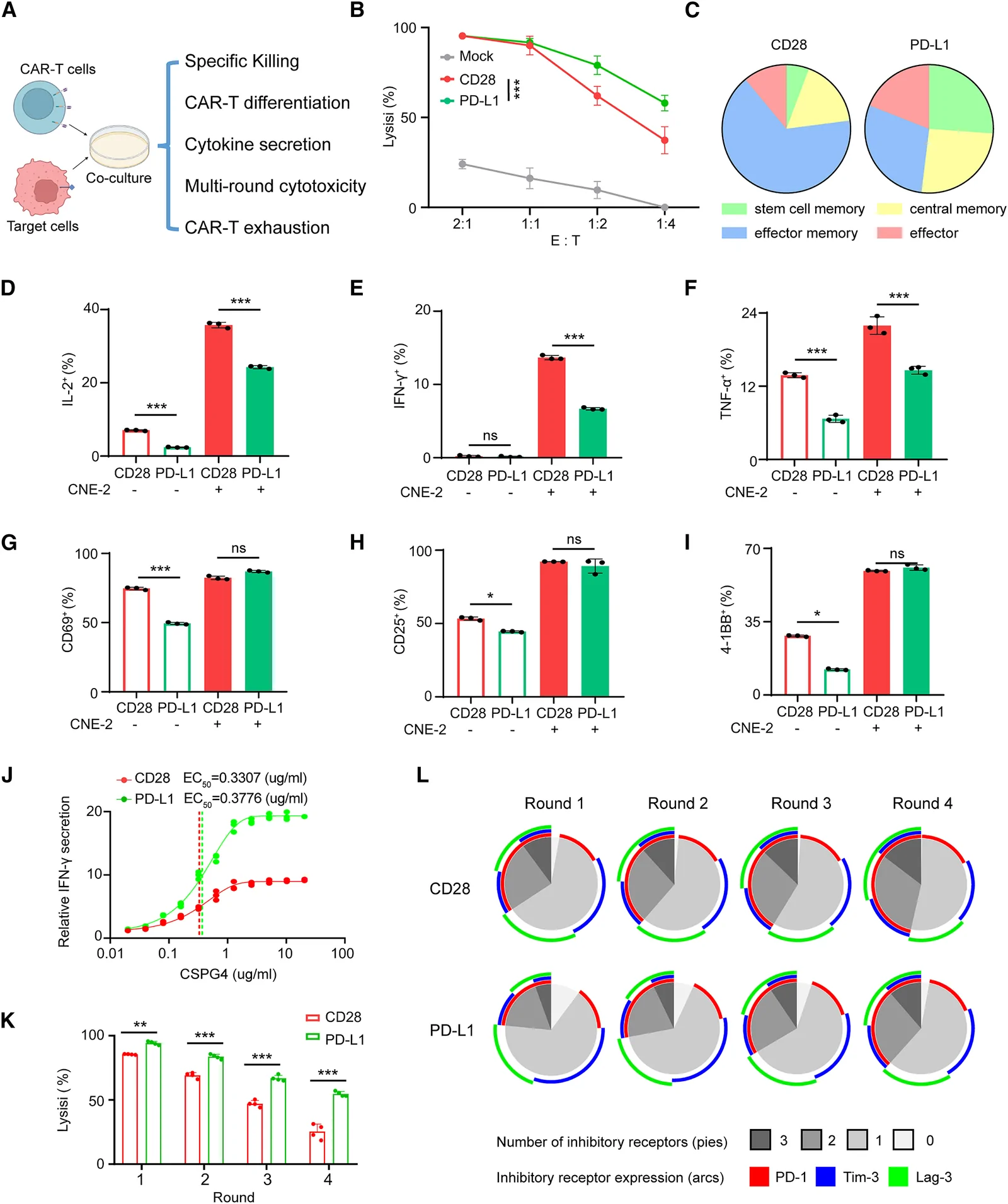
PD-L1 TM CAR-T cells exhibit superior and sustained tumor cell killing *in vitro* (A) Schematic of the experimental workflow. Human T cells were activated with CD3/CD28 Dynabeads and transduced 48 h later with lentivirus encoding a second-generation CSPG4 CAR. Single-round killing, differentiation, and cytokine secretion assays were performed on day 7 post-transduction, and the multi-round cytotoxicity assay was conducted from day 7 to day 12. (B) Cytotoxicity of CSPG4 CAR-T cells against CNE-2 target cells measured by firefly luciferase (FFLuc) reporter assay at the indicated effector-to-target (E:T) ratios after 24 h co-culture. ∗∗∗*p* < 0.001. Data represent the mean ± SEM of *n* = 3 biologically independent experiments. (C) Memory phenotype of CSPG4 CAR-T cells 24 h after stimulation with CNE-2 cells at a 1:1 E:T ratio. Tscm, CD45RA^+^CD62L^+^; Tcm, CD45RA^−^CD62L^+^; Tem, CD45RA^−^CD62L^−^; Teff, CD45RA^+^CD62L^−^. (D–F) Intracellular cytokine staining for IL-2 (D), IFN-γ (E), and TNF-α (F) in CSPG4 CAR-T cells before and after 8 h of stimulation with CNE-2 cells. Data represent the mean ± SEM of *n* = 3 biologically independent experiments. (G–I) Expression of the activation markers CD69 (G), CD25 (H), and 4-1BB (I) on CSPG4 CAR-T cells before and after 24 h of stimulation with CNE-2 cells. Data represent the mean ± SEM of *n* = 3 biologically independent experiments. (J) Antigen sensitivity determined by titration of plate-coated recombinant CSPG4 protein with IFN-γ ELISA readout. EC50, half maximal effective concentration. Data represent the mean ± SEM of *n* = 3 biologically independent experiments. (K) Cytolytic activity of CSPG4 CAR-T cells against live CNE-2 cells after the 1st to 4th rounds of stimulation with irradiated CNE-2 cells, assessed by a 24-h FFLuc reporter assay at a 1:1 E:T ratio. Data represent the mean ± SEM of *n* = 4 biologically independent experiments. (L) Co-expression of PD-1, Lag-3, and Tim-3 on CSPG4 CAR-T cells at each round of stimulation.

We next examined the differentiation state of CAR-T cells 24 h after tumor challenge. In both CSPG4 and GD2 systems, PD-L1 TM CAR-T cells retained significantly higher proportions of Tscm and Tcm cells than CD28 TM CAR-T cells (Figures 3C and S5E). Cytokine profiling revealed a restrained secretory phenotype in PD-L1 TM CAR-T cells, consistent with previous reports that monomeric CAR designs generally exhibit reduced cytokine secretion.^46^ At rest, they produced lower levels of IL-2, IFN-γ, and TNF-α, and this difference persisted following antigen stimulation across both CAR specificities (Figures 3D–3F and S5F–S5H). Despite their lower basal activation state, PD-L1 TM CAR-T cells strongly upregulated CD69, CD25, and 4-1BB upon tumor encounter, reaching levels comparable to or exceeding those of CD28 TM CAR-T cells (Figures 3G–3I and S5I–S5K). This pattern of low resting tone with robust antigen-inducible activation suggests a healthier, less exhaustion-prone activation program. To exclude the possibility that reduced tonic signaling compromises antigen sensitivity, we determined the functional affinity of CSPG4 CARs by titrating plate-coated recombinant antigen. The EC50 values were highly similar between CD28 and PD-L1 TM CAR-T cells, while the relative fold change in IFN-γ secretion from baseline to maximal stimulation was substantially greater for the PD-L1 TM group (Figure 3J), reflecting an improved signal-to-noise ratio.

Given the critical importance of sustained function under chronic antigen stimulation without exhaustion for effective T cell-based cancer immunotherapies,^47^ we then evaluated functional persistence using a repetitive antigen stimulation assay. Both CSPG4 and GD2 CAR-T cells underwent four successive rounds of co-culture with live target cells, and cytotoxicity was assessed after each round (Figure S5L). PD-L1 TM CAR-T cells maintained significantly higher killing capacity than CD28 TM CAR-T cells across all four stimulations (Figures 3K and S5M). Furthermore, PD-L1 TM CAR-T cells showed markedly lower expression of PD-1, Lag-3, and Tim-3 (Figures 3L and S5N–S5P), accompanied by higher TCF1 and CCR7 expression (Figures S5Q and S5R), indicating reduced exhaustion accumulation and preserved stemness under chronic antigen pressure. Collectively, these results establish that the PD-L1 TM swap generates CAR-T cells with enhanced and sustained antitumor cytotoxicity and a favorable, exhaustion-resistant functional profile across multiple CAR specificities.

### PD-L1 TM domain enhances antitumor efficacy and promotes T cell persistence *in vivo*

To evaluate the therapeutic potential of PD-L1 TM CAR-T cells *in vivo*, we established a subcutaneous xenograft model using a CSPG4-expressing tumor cell line (Figure 4A). Consistent with the *in vivo* findings reported by Dotti and colleagues, CD28 TM CAR-T cells failed to control tumor growth, showing tumor burdens comparable to those of the mock group.^48^ In contrast, PD-L1 TM CAR-T cells significantly enhanced tumor control, as evidenced by reduced tumor growth over time, smaller resected tumors, and decreased final tumor weights (Figures 4B–4D). PD-L1 TM CAR-T cells also exhibited significantly higher frequencies in peripheral blood within both the CD8^+^ and CD4^+^ compartments (Figures 4E and 4F). Immunofluorescence analysis of tumor sections revealed enhanced CAR-T cell infiltration and reduced tumor cell proliferation (Ki67) in the PD-L1 TM group (Figure 4G). Consistent with this, flow cytometry confirmed increased proportions of CD8^+^ and CD4^+^ CAR-T cells in both tumor tissues (Figures 4H and 4I) and spleens (Figures 4J and 4K) of mice treated with PD-L1 TM CAR-T cells. Furthermore, splenic CAR-T cells from the PD-L1 TM group contained significantly enriched Tscm and Tcm populations (Figure 4L).

**Figure 4:**
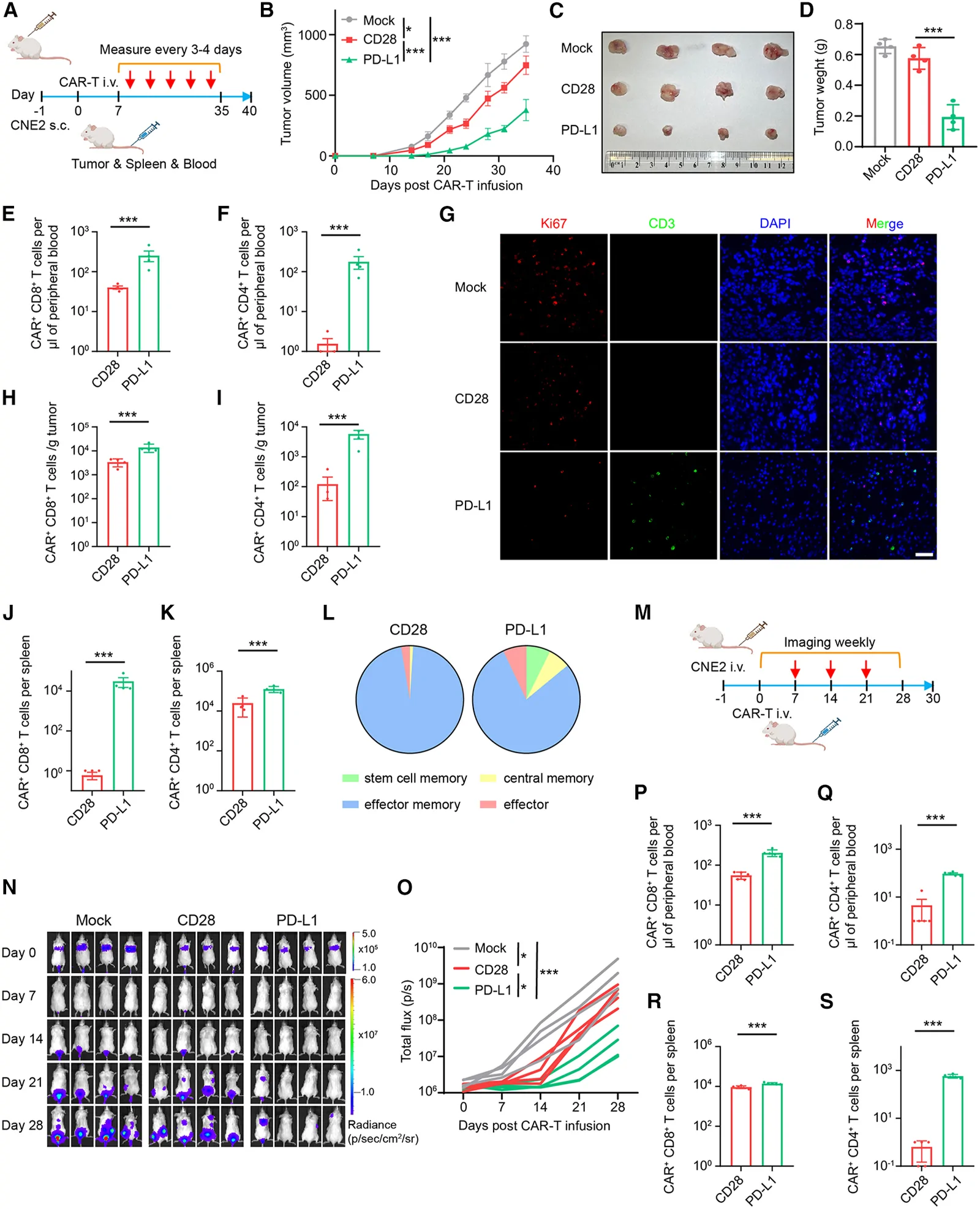
PD-L1 TM domain enhances antitumor efficacy and promotes T cell persistence *in vivo* (A) Schematic of the subcutaneous CNE-2 xenograft model. (B) Tumor growth curves over time following CAR-T cell administration (*n* = 4 per group). *p* values were determined by two-way ANOVA. (C) Representative photographs of resected tumors on day 40. (D) Tumor weights of excised CNE-2 tumors at endpoint. *p* values were determined by unpaired Student’s *t* test. (E and F) Absolute counts of CAR^+^CD8^+^ (E) and CAR^+^CD4^+^ (F) T cells per μL peripheral blood on day 40. ∗∗∗*p* < 0.001. For two-group comparisons, we used Hedges’s g with 95% confidence intervals (*n* < 20). Hedges’s g values for (E) and (F) were 1.728 and 1.746, respectively. (G) Representative immunofluorescence images of tumor sections stained for Ki67 and CD3. Scale bar, 50 μm. (H and I) Absolute counts of CAR^+^CD8^+^ (H) and CAR^+^CD4^+^ (I) T cells per g tumor tissue on day 40, determined by flow cytometry after enzymatic digestion. ∗∗∗*p* < 0.001. For two-group comparisons, we used Hedges’s g with 95% confidence intervals (*n* < 20). Hedges’s g values for (H) and (I) were 2.376 and 1.862, respectively. (J and K) Absolute counts of CAR^+^CD8^+^ (J) and CAR^+^CD4^+^ (K) T cells per spleen on day 40. ∗∗∗*p* < 0.001. For two-group comparisons, we used Hedges’s g with 95% confidence intervals (*n* < 20). Hedges’s g values for (J) and (K) were 1.201 and 2.650, respectively. (L) Memory subset distribution of splenic CAR-T cells on day 40. Tscm, CD45RA^+^CD62L^+^; Tcm, CD45RA^−^CD62L^+^; Tem, CD45RA^−^CD62L^−^; Teff, CD45RA^+^CD62L^−^. (M) Schematic of the disseminated xenograft model. NSG mice were injected intravenously with CSPG4-expressing tumor cells and 1 day later received CAR-T cells. Tumor burden was monitored weekly by bioluminescence imaging. Mice were sacrificed on day 30. (N) Representative bioluminescence images on the indicated days. (O) Quantification of bioluminescence signal (N) over time. (P and Q) Absolute counts of CAR^+^CD8^+^ (P) and CAR^+^CD4^+^ (Q) T cells per μL of peripheral blood on day 30. ∗∗∗*p* < 0.001. For two-group comparisons, we used Hedges’s g with 95% confidence intervals (*n* < 20). Hedges’s g values for (P) and (Q) were 4.691 and 6.302, respectively. (R and S) Absolute counts of CAR^+^CD8^+^ (R) and CAR^+^CD4^+^ (S) T cells per spleen on day 30. ∗∗∗*p* < 0.001. For two-group comparisons, we used Hedges’s g with 95% confidence intervals (*n* < 20). Hedges’s g values for (R) and (S) were 2.853 and 7.256, respectively.

We further validated these findings in a disseminated tumor model (Figure 4M), in which PD-L1 TM CAR-T cells achieved significantly stronger suppression of tumor progression than CD28 TM CAR-T cells, as assessed by bioluminescence imaging (Figures 4N and 4O). Consistently, PD-L1 TM CAR-T cells displayed enhanced persistence in both peripheral blood and spleen in this metastatic setting (Figures 4P–4S). To determine whether these benefits extend beyond the CSPG4 CAR system, we evaluated GD2-specific CAR-T cells in a disseminated Nalm6-GD2 tumor model (Figure S6A), a well-established system widely used to assess the *in vivo* efficacy of GD2 CAR-T cells.^38^^,^^39^^,^^40^ Consistent with previous reports using this model, CD28 TM GD2 CAR-T cells failed to control tumor growth, with tumor burden comparable to that of controls. PD-L1 TM GD2 CAR-T cells again showed superior tumor control and significantly increased persistence in both blood and spleen compared with CD28 TM GD2 CAR-T cells (Figures S6B–S6F). Collectively, these *in vivo* results demonstrate that the PD-L1 TM domain not only enhances antitumor efficacy in both localized and disseminated tumor models but also promotes CAR-T cell persistence and memory maintenance, underscoring its broad therapeutic potential to improve CAR-T cell therapy outcomes.

### PD-L1 TM domain confers an exhaustion-resistant, stemness- and memory-enriched transcriptional profile

To understand how the PD-L1 TM attenuates tonic signaling at the molecular level, we examined the phosphorylation status of key signaling nodes downstream of CD3ζ, including the immediate kinase ZAP70, the adaptor SLP-76, the second messenger enzyme PLC-γ1, and the terminal effectors AKT, NF-κB, and ERK. The degree of phosphorylation at each node followed a consistent hierarchy, with CD28 TM CAR-T cells exhibiting the highest levels, PD-L1 TM CAR-T cells showing intermediate levels, and the Beads control displaying the lowest (Figure 5A). This uniform reduction across the entire CD3ζ-ZAP70-PLC-γ1/MAPK/AKT/NF-κB axis indicates that the PD-L1 TM suppresses tonic signaling from the very proximal step and that this suppression is faithfully transmitted through all major downstream branches.

**Figure 5:**
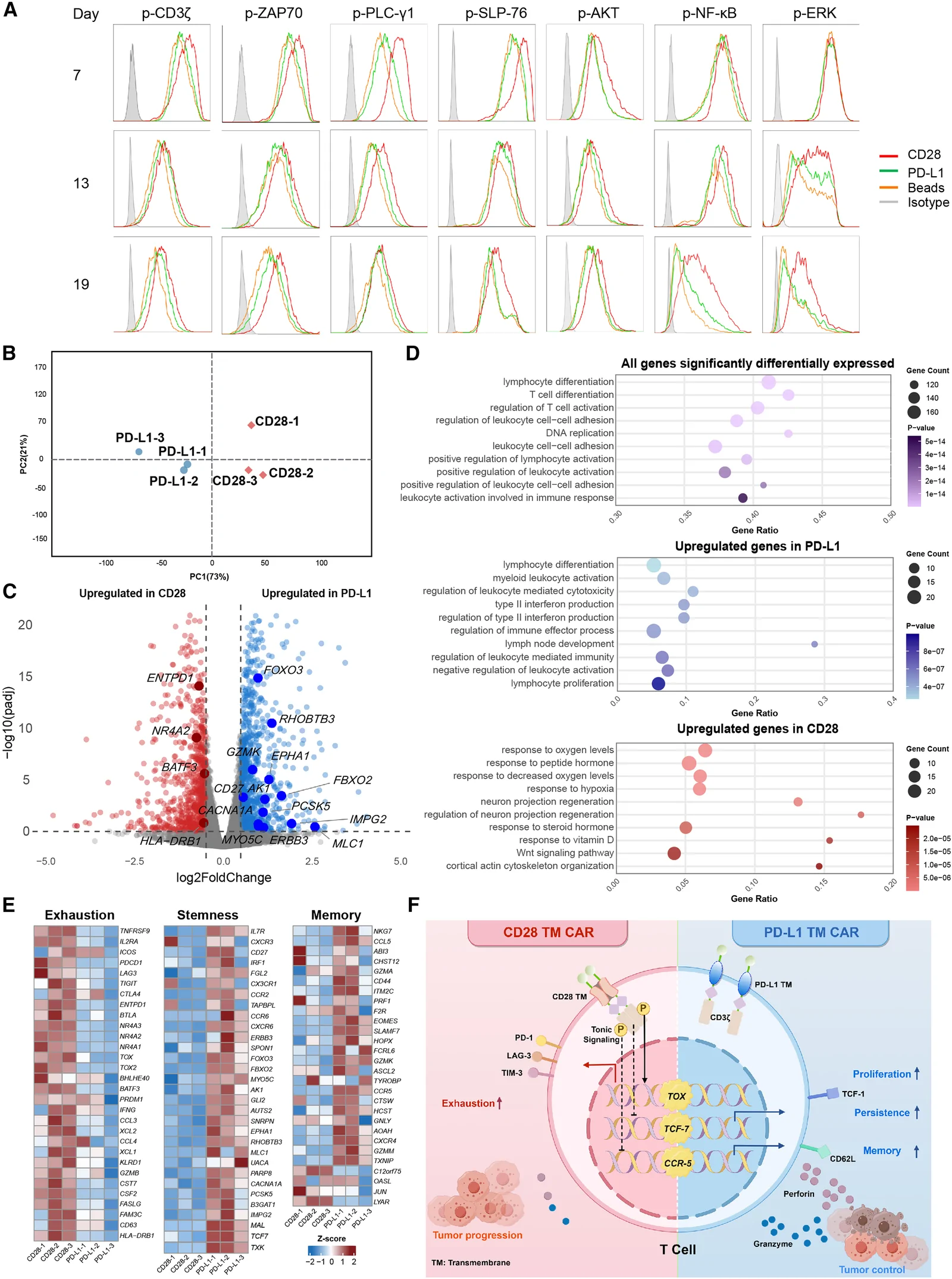
PD-L1 TM domain confers an exhaustion-resistant, stemness- and memory-enriched transcriptional profile (A) Human T cells were activated with CD3/CD28 Dynabeads and transduced 48 h later with lentivirus encoding a second-generation CSPG4 CAR. Phospho-protein flow cytometry was performed on days 7, 13, and 19 post-transduction. (B) Bulk RNA-seq was performed on day 14 post-transduction. Principal component analysis of CD28 TM and PD-L1 TM CSPG4 CAR-T cell transcriptomes, showing separation between the two groups. (C) Volcano plot of differentially expressed genes. The *x* axis represents fold change (PD-L1 vs. CD28), and the *y* axis represents statistical significance. (D) Gene Ontology analysis of differentially expressed genes. (E) mRNA levels of exhaustion-, stemness-, and memory-related genes in PD-L1 TM vs. CD28 TM CSPG4 CAR-T cells. (F) Schematic model illustrating how TM domain engineering calibrates CAR tonic signaling.

To extend these findings to the transcriptional level, we performed bulk RNA sequencing (RNA-seq) on day 14 CAR-T cell products. Principal component analysis revealed pronounced transcriptional separation between PD-L1 TM and CD28 TM CSPG4 CAR-T cells, indicating fundamentally distinct cellular states (Figure 5B). Unsupervised differential expression analysis identified numerous significantly altered genes, with PD-L1 TM CAR-T cells showing marked upregulation of *FOXO3*, *FBXO2*, and *EPHA1*, alongside significant downregulation of exhaustion-associated transcripts including *ENTPD1*, *BATF3*, and *NR4A2* (Figure 5C). Gene Ontology analysis of differentially expressed genes revealed enrichment in lymphocyte differentiation, T cell activation, and immune response pathways. Genes upregulated in PD-L1 TM CAR-T cells were specifically associated with lymph node development, type II interferon production, and immune effector regulation, whereas CD28 TM CAR-T cells showed preferential enrichment in stress-response pathways, including hypoxia, peptide hormone response, and Wnt signaling (Figure 5D). Comprehensive profiling of exhaustion-, stemness-, and memory-related genes revealed that PD-L1 TM CAR-T cells significantly downregulated key exhaustion mediators (*PDCD1*, *LAG3*, *TIGIT*, *CTLA4*, *ENTPD1*, and multiple *NR4A* family members) while upregulating stemness-associated factors (*TCF7*, *FOXO3*, *CD27*, and *CXCR3*) and memory-related effectors (*GNLY*, *GZMA*, *GZMK*, *CCR5*, and *CXCR4*) (Figure 5E).

We validated these transcriptional patterns in the GD2 CAR system, where the PD-L1 TM similarly reduced exhaustion and apoptosis signatures while enhancing memory programs (Figures S7A–S7D). Based on these findings, we propose a model in which TM domain optimization through PD-L1 TM incorporation represents a rational strategy to calibrate CAR signaling (Figure 5F). This minimal structural modification suppresses spontaneous receptor clustering, attenuates tonic signaling, and establishes a transcriptional program favoring long-term persistence and antitumor efficacy. Our work establishes PD-L1 TM as an optimal architecture that enhances CAR-T cell fitness by reducing exhaustion and promoting stemness and memory, offering a straightforward yet powerful strategy to advance solid tumor immunotherapy.

## Discussion

Tonic signaling, referring to the antigen-independent, low-level constitutive signaling of immune antigen receptors, is indispensable for T cell survival, development, proliferation, and differentiation.^33^^,^^48^^,^^49^ However, elevated tonic signaling can drive premature effector differentiation in CAR-T cells, thereby impairing their long-term antitumor efficacy.^50^^,^^51^ The impact of tonic signaling on CAR-T efficacy is best understood as a bell-shaped relationship. Insufficient tonic signaling compromises T cell proliferation and *in vivo* persistence, whereas excessive signaling accelerates differentiation and exhaustion, ultimately undermining cytotoxic durability.^12^^,^^17^^,^^52^^,^^53^ Furthermore, interventions that extend the duration of CAR signaling at the membrane increase persistence for weak-signaling CARs, further illustrating that both signal strength and duration determine therapeutic outcomes.^54^ For solid tumor targets, many clinically relevant CAR constructs inherently exhibit high tonic signaling, positioning them on the deleterious right side of this curve.^15^^,^^17^^,^^39^^,^^40^^,^^55^^,^^56^ Therefore, strategies that reduce excessive tonic signaling without abolishing antigen responsiveness are particularly desirable.

Recent studies have further refined our understanding of tonic signaling regulation at multiple levels. At the scFv level, arginine residue introduction enhances CAR self-interaction and antitumor efficacy,^57^ while linker length modulation can decouple binding affinity from tonic signaling.^58^ At the intracellular level, alternative CD3 subunits such as CD3ε can serve as low-tonic signaling modules,^59^ and the transcriptional regulator BACH2 has been identified as a key mediator that directs stem and memory programs downstream of tonic signaling.^60^ Dynamic regulation strategies have also emerged, including drug-inducible phase-separation CARs and activation-induced release motifs that provide cell-autonomous control of surface CAR expression.^61^^,^^62^ Modifications to hinge length, costimulatory domain selection, and CD3ζ ITAM configuration have similarly been shown to modulate tonic signaling intensity and functional persistence.^16^^,^^23^^,^^47^^,^^59^^,^^63^ Despite these advances, the TM domain has remained relatively overlooked as an active contributor to tonic signaling, despite its fundamental role in mediating helix-helix interactions, receptor assembly, and membrane organization.^64^ Our study addresses this gap by demonstrating that TM sequence identity alone can significantly influence CAR clustering and tonic signaling, establishing the TM domain as a previously underexploited yet readily targetable design parameter.

The CD28 and CD8α TMs commonly used in CARs contain dimerization motifs, such as the GxxxA motif, that promote helix-helix interactions and spontaneous receptor clustering, contributing to elevated tonic signaling.^25^^,^^65^^,^^66^^,^^67^ In contrast, the PD-L1 TM domain lacks these canonical dimerization motifs and instead supports a predominantly monomeric conformation. Additionally, torsional reorientation analysis revealed that the PD-L1 TM produces a 218° rotation relative to CD28 TM (Figure 1H), a configuration previously shown to enhance CAR function.^34^ In addition, the PD-L1 TM displays the smallest tilt angle and lowest hydrophobic thickness among the TMs tested (Figure 1I), enabling a better match with the lipid bilayer that minimizes hydrophobic mismatch-driven clustering.^35^^,^^36^ Together, these biophysical properties explain how this single TM substitution achieves significant tonic signaling reduction without altering antigen recognition or intracellular signaling competence.

Tonic signaling-driven exhaustion, arising from chronic antigen-independent CAR signaling, is characterized by spontaneous ligand-independent CAR clustering that drives upregulation of inhibitory receptors and transcriptional changes, which can be reliably detected within 1–2 weeks in both GD2 and CSPG4 CAR-T cell models.^15^^,^^17^^,^^31^^,^^38^^,^^39^^,^^40^^,^^47^ Our finding that PD-L1 TM substitution mitigates this exhaustion trajectory aligns with and extends several strategies that counteract tonic signaling-induced exhaustion, including c-Jun overexpression,^40^ drug-inducible transient rest,^39^ metabolic interventions,^38^^,^^68^ epigenetic reprogramming,^69^^,^^70^^,^^71^^,^^72^ and immunological synapse remodeling.^73^^,^^74^ Beyond these T cell-intrinsic approaches, cell-based immunomodulatory strategies such as cytokine-licensed mesenchymal stromal cells (MSCs) also were able to temper excessive T cell activation.^75^^,^^76^ Unlike these downstream or extrinsic interventions, the PD-L1 TM swap acts directly at the receptor level by reducing CAR oligomerization propensity, thereby attenuating tonic signaling at its most proximal source. This mechanism is fundamentally distinct from T cell receptor alpha constant (TRAC) locus integration, which reduces tonic signaling through physiological CAR expression levels.^70^ By targeting the structural basis of spontaneous CAR clustering, TM engineering offers a structurally minimal, non-genomic strategy that may synergize with transcriptional, metabolic, or even extrinsic cellular immunomodulatory interventions to achieve even greater functional preservation.

Parallel to exhaustion mitigation, PD-L1 TM CAR-T cells are enriched for Tscm and Tcm phenotypes, which support self-renewal, lymphoid homing, and long-term persistence. We defined Tscm cells by the CD45RA^+^CD62L^+^ phenotype, which reflects the re-expression of CD45RA in a subset of activated T cells that retain memory features, as originally established by Gattinoni et al. and now widely adopted in the CAR-T field.^41^^,^^69^ Mechanistically, the attenuated tonic signaling resulting from PD-L1 TM incorporation may help preserve chromatin accessibility and transcriptional states conducive to memory maintenance rather than promoting terminal effector differentiation. This aligns with manufacturing strategies that leverage naive or stem memory T cells as starting populations, which exhibit superior persistence and a reduced propensity for exhaustion and cytokine-related toxicity.^77^ It also corroborates the observation that costimulatory cues such as CD81 can skew T cell activation toward naive-like trajectories, enriching products with enhanced durability potential even before antigen encounter.^78^ By shifting the CAR-T product toward a memory-biased composition at infusion, TM domain optimization promotes sustained engraftment and long-term persistence, an effect similarly observed in stem cell-derived CAR-T systems.^79^

Despite these encouraging results, several limitations merit emphasis. First, the CNE-2 cell line used in our xenograft models has been reported to carry a risk of HeLa cell contamination. However, its robust CSPG4 expression was validated (Figure S5A); therefore, this potential contamination does not compromise its feasibility and value as a CSPG4-positive target cell for evaluating CSPG4-directed CAR-T cells. Second, the Nalm6-GD2 model, which is widely employed for GD2 CAR-T cell evaluation both *in vitro* and *in vivo*, is a hematopoietic model that lacks the stromal barriers and immunosuppressive microenvironment of solid tumors and thus primarily captures cell-intrinsic effects on persistence and exhaustion resistance. Finally, further validation across a broader panel of CAR specificities will be needed to establish the generalizability of the PD-L1 TM strategy.

In summary, our data demonstrate that TM domain selection is a simple yet powerful strategy to tune CAR tonic signaling, reshape transcriptional programs, and enhance functional persistence. Substituting the dimerization-prone CD28 TM with the PD-L1 TM generates CAR-T cells with reduced exhaustion, enhanced memory commitment, and superior durability under repeated antigen challenge. By directly addressing the elevated tonic signaling often inherent to solid tumor-targeting CARs, TM engineering offers a structurally minimal and broadly applicable solution that complements existing optimization strategies.

## Materials and methods

### Reagents and antibodies

The following reagents were purchased from the indicated suppliers: LIVE/DEAD Fixable Near-IR Dead Cell Stain Kit (L34975) from Invitrogen, GMOne-Step Luciferase Reporter Gene Assay Kit (GM-040503A) from Genomeditech, D-luciferin (122799) from PerkinElmer, HumanT-Activator CD3/CD28 Dynabeads (11132D) from Life Technologies, recombinant human IL-2 (GMP-CD66) from Novoprotein, and Recombinant Human CSPG4 protein (P0598) from Fine Test. The following antibodies were used for fluorescence-activated cell sorting (FACS): anti-CD69-APC (BioLegend, 310910), anti-CD25-APC (BioLegend, 302610), anti-ICOS-APC (BioLegend, 313510), anti-PD-1-APC (BioLegend, 329908), anti-LAG-3-PE-Cy7 (BioLegend, 369208), anti-TIM-3-APC (BioLegend, 364804), anti-CD62L-APC (BioLegend, 385106), anti-CD45RA-PE-Cy7 (BioLegend, 304126), anti-CCR7-PE (BioLegend, 353204), anti-TCF1/TCF7-PE (BioLegend, 655207), anti-CD8a-PE-Cy7 (BioLegend, 344712), anti-CD4-APC (BioLegend, 344614), anti-phospho-ZAP70-PE-Cy7 (Invitrogen, 2833613), anti-Granzyme B-APC (BioLegend, 372204), anti-4-1BB-APC (BioLegend, 309810), anti-TNF-α-APC (BioLegend, 502912), anti-IFN-γ-PE-Cy7 (BioLegend, 502528), anti-IL-2-APC (BioLegend, 500310), anti-TOX-PE (Thermo Fisher Scientific, 12-6502-82). anti-Phospho-CD3ζ(Tyr142) (CST, 67748), anti-Phospho-ZAP70 (Tyr319)/Syk (Tyr352) (65E4) (CST, 2717), anti-Phospho-SLP-76 (Ser376) (D9D6E) (CST, 14745), anti-Phospho-PLC-γ1 (Tyr783) (CST, 2821), anti-Phospho-Akt (Ser473) (CST, 9271S), anti-phospho-NF-κB p65 (Ser536) (CST, 3033S), anti-p44/42 MAPK (Erk1/2) (CST, 9102S), and anti-Phospho-p44/42 MAPK (Erk1/2) (Thr202/Tyr204) (CST, 9101S). For confocal microscopy, the following antibodies were used: anti-hIgG-Alexa Fluor 647 (Jackson, 109-605-098), Ki67-Alexa Fluor Plus 555 (Invitrogen, 740008TP55520UG), CD3 (Proteintech, 17617-1-AP), and DAPI (Beyotime, C1341).

### Cell lines

The cell lines used in this study were obtained from the following sources: K562 (ATCC, CCL-243) and HEK293T (Chinese Academy of Sciences Cell Bank, SCSP-502). Jurkat, CNE-2, K562-CD19, K562-CSPG4, K562-mesothelin, and Nalm6-GD2 cells were cultured in RPMI 1640 medium (Gibco, 11875093), while HEK293T and 143B cells were maintained in Dulbecco’s modified Eagle’s medium (DMEM; Gibco, C11995500BT). All media were supplemented with 10% fetal bovine serum (FBS; Gibco, A5669701) and 1% penicillin/streptomycin (Gibco, 15140122). The cells were incubated at 37°C in a humidified atmosphere containing 5% CO_2_.

### Lentivirus vector construction and virus preparation

A second-generation CAR targeting CSPG4, GD2, and mesothelin was constructed using the pWPXLd lentiviral vector (Addgene, #12258), with expression driven by the EF1a promoter. The CAR construct consisted of an scFv region, a hinge and TM domain, and the CD28 and CD3ζ endodomains, which were fused to an IRES-GFP reporter gene. Lentiviral particles were produced in HEK293T cells by co-transfecting 5 × 10^6^ cells with the transfer vector and the packaging plasmids pSPAX2 (Addgene, #12260) and pMD2.G (Addgene, #12259) using PEI transfection reagent (life-ilab, AC04L098). The viral supernatant was collected 48 h post-transfection, filtered, and concentrated with the Lenti-X Concentrator (Takara, 631232) before being stored at −80°C.

### Jurkat cells lentiviral transduction

Jurkat cells were transduced via spinfection at 800 × *g* for 90 min at 32°C with lentivirus at an MOI of 4, in the presence of 0.8 μg/mL polybrene (Beyotime, C0351). Following transduction, the cells were maintained in RPMI-1640 medium supplemented with 10% FBS and 1% penicillin-streptomycin.

### T cell activation and culturing

Cryopreserved human T lymphocytes from healthy donors were thawed on day 0 and activated with CD3/CD28 Dynabeads at a 3:1 bead-to-cell ratio. The activated T cells were then cultured in T75 flasks at a density of 0.5–1 × 10^6^ cells/mL using X-VIVO medium (Lonza, BE02-054Q) supplemented with 10% FBS, 1% penicillin-streptomycin, and 100 IU/mL recombinant human IL-2.

### Primary T cell lentiviral transduction

Activated primary human T cells were transduced via spin infection (800 × *g*, 90 min, 32°C) using lentivirus at an MOI of 10 in the presence of 0.8 μg/mL polybrene. Following transduction, the cells were maintained in X-VIVO medium supplemented with 5% FBS, 1% penicillin-streptomycin, and 100 IU/mL recombinant human IL-2.

### Flow cytometry analysis

Cell surface staining was performed by incubating cells with antibodies for 30 min at 4°C in the dark. For intracellular targets, cells were first fixed and permeabilized using the FoxP3 Transcription Factor Staining Buffer Set (Thermo Fisher Scientific, 00-5523-00) prior to a 1-h antibody incubation at room temperature. Viable cells were discriminated using the LIVE/DEAD Fixable Near-IR Dead Cell Stain Kit. Cell sorting was conducted on a FACSAria III cytometer, while data acquisition was performed on an LSRFortessa flow cytometer and analyzed using FlowJo v.10 software.

### Intracellular phosphoprotein analyses

CAR-T cells were first stained with Fixable Viability Dye eFluor 506 (eBioscience, 65-0866-14) for 30 min at 4°C in the dark to discriminate dead cells. After washing with PBS, cells were fixed with 4% paraformaldehyde (PFA) for 15 min at room temperature, washed twice with PBS, and permeabilized with ice-cold 90% methanol for 30 min on ice. Following permeabilization, cells were washed twice with PBS containing 2% FBS and stained with phospho-specific antibodies for 1 h at room temperature in the dark. After a final wash, samples were resuspended in PBS and acquired on an LSRFortessa flow cytometer. Data were analyzed using FlowJo v.10 software.

### Confocal microscopy

To analyze CAR surface clustering, Jurkat cells expressing the relevant CARs were fixed with 4% PFA and blocked with 5% BSA in PBS. CAR clusters were visualized using an APC-conjugated anti-human IgG antibody (1 μg/mL; Jackson Immuno Research, 109-605-098). For evaluation of Ki67 and CD3 expression in CSPG4 CAR xenograft tissues, samples were fixed in 10% neutral buffered formalin, dehydrated, and embedded in paraffin. Antigen retrieval was performed by heating sections in sodium citrate buffer (pH 6.0) using a pressure cooker for 3 min. After permeabilization with 0.1% Triton X-100 in PBS, sections were blocked with 5% BSA and incubated with primary antibodies (anti-Ki67 or anti-CD3) for 2 h at room temperature. Following PBS washes, sections were incubated with appropriate secondary antibodies for 30 min, counterstained with DAPI, and imaged using a Leica TCS SP8 confocal microscope. Image analysis was conducted with ImageJ software.

### BiFC assay

Jurkat cells were seeded into 12-well plates at 0.5 × 10^6^ cells per well and cultured overnight. Cells were co-transfected using Lipo DC MAX (Genvaxell K204) with 0.5 μg each of pBiFC-VN155(I152L) and pBiFC-VC155 plasmids, both encoding the same CSPG4 CAR sequence with the split Venus fragments fused to the intracellular tail. After 24 h, cells were fixed with 4% PFA and imaged by fluorescence microscopy. Images were analyzed using ImageJ software. For flow cytometric quantification, cells were washed with PBS and analyzed on a flow cytometer.

### Prediction and membrane positioning of CAR TM domains

The full-length amino acid sequences of the four CAR constructs were submitted to DeepTMHMM v.1.0 (https://services.healthtech.dtu.dk/services/DeepTMHMM-1.0/) to predict the TM topology and define the TM-core boundaries.^80^ For each construct, the predicted TM sequence together with 15 flanking amino acids on both the N- and C-terminal sides was modeled using the SWISS-MODEL server (https://swissmodel.expasy.org/).^81^ The resulting structural models were submitted in PDB format to the PPM server (https://opm.phar.umich.edu/ppm_server) to determine the preferred membrane orientation and obtain the predicted TM segment, membrane-embedded residues, tilt angle, hydrophobic thickness, and water-to-membrane transfer free energy.^82^

### *In vitro* cytotoxicity assay

The cytotoxic activity of CAR-T cells was evaluated using a luciferase-based killing assay. Firefly luciferase-expressing target cells (1 × 10^4^ per well) were co-cultured with CAR-T cells at various effector-to-target (E:T) ratios in 96-well plates for 24 h at 37°C. Following co-culture, 100 μL of GMOne luciferase substrate was added to each well in a white luminometer plate. Luminescence was quantified using a PerkinElmer Enspire plate reader. The specific killing percentage was calculated by normalizing the luminescence in co-culture wells to that of target cells cultured alone.

### Repeated antigen stimulation assay

A repeated stimulation assay was conducted over 4 days. Briefly, 5 × 10^6^ CAR-T cells were seeded into a 10 cm dish with T cell medium, and 1× 10^6^ irradiated target tumor cells were added as stimulators. Irradiation was used to prevent target cell proliferation while maintaining their antigen-presenting function during the stimulation period. At each 24-h time point, CAR-T cells were collected from the co-culture and moved to a new well. A small sample was removed for flow cytometry and killing assay, and the remaining CAR-T cells were centrifuged. After discarding part of the supernatant to retain 50% of the original volume, a fresh dose of irradiated 1 × 10^6^ target tumor cells in T cell medium was supplied at the same volume. This feeding cycle was repeated to complete a 4-day culture period.

### Functional affinity assay

CSPG4 CAR-T cells were seeded into 96-well plates pre-coated with graded concentrations of recombinant CSPG4 protein. After 18 h of stimulation, human IFN-γ levels in the culture supernatants were quantified by ELISA.

### RNA-seq

RNA-seq libraries were generated from 1 × 10^6^ isolated CAR-T cells using a modified SMART-Seq2 method. Briefly, total RNA was extracted, and 100 ng of it was hybridized with a TSO oligo-dT primer (5′-AAGCAGTGGTATCAACGCAGAGTACT30VN-3′). Next, reverse transcription was performed using the Template Switching RT Enzyme Mix (NEB, M0466S), followed by cDNA pre-amplification for 8 cycles with Q5 High-Fidelity DNA Polymerase (NEB, M0491S). The amplified cDNA was purified with AMPure XP beads (Beckman Coulter, A63880) and tagmented using Tn5 transposase. Final libraries were amplified by PCR and sequenced on an Illumina NovaSeq system. Reads were aligned to the GRCh37/hg19 genome with STAR v.2.7.5a, and gene counts were generated by HTSeq v.0.12.4. Quality control metrics showed total read counts of 20–30 million paired-end reads per sample, with alignment rates exceeding 90% and over 15,000 genes detected per sample. Data were merged and normalized using a custom R script and DESeq2. Heatmaps were visualized with OmicStudio tools.

### Mouse experiments

Five- to eight-week-old NOD/SCID/Il2rg-null (NSG) mice from Biocytogen (#110586) were used in the mouse experiments. For the anti-CSPG4 subcutaneous xenograft tumor model using CNE2 cells, mice were inoculated with 1 × 10^5^ luciferase-expressing tumor cells 1 day before adoptive cell transfer of 5 × 10^6^ CAR-T or non-transduced T cells intravenously. Tumor cells were injected subcutaneously into the right flank at the midpoint between the axilla and the inguinal region. For the anti-CSPG4 metastasis model using CNE2 cells, mice were inoculated with 5 × 10^4^ luciferase-expressing tumor cells 1 day before the adoptive transfer of 6 × 10^6^ CAR-T or non-transduced T cells intravenously. For anti-GD2 xenograft experiments using Nalm6-GD2 cells, mice were inoculated intravenously with 2.5 × 10^5^ luciferase-expressing tumor cells 3 days prior to the adoptive transfer of 5 × 10^6^ CAR-T or non-transduced T cells. Tumor progression was monitored by bioluminescence imaging using the IVIS spectrum imaging system (PerkinElmer). Mice were euthanized upon signs of excessive tumor burden, and survival curves were generated accordingly. Spleen and peripheral blood samples were collected at the indicated time points to evaluate CAR-T cell persistence by flow cytometry. The xenograft tissues were measured in size (mm^3^) with the formula 0.5 × (length × width × width) and weighed. After sacrificing the animals, the xenograft tissues were then fixed in 4% PFA and embedded in paraffin. Paraffin sections were subjected to immunofluorescence staining. All animal experiments were approved by our animal ethical committee (IACUC number: 20250110002).

### Statistical analysis

The data are presented as mean ± SEM from a minimum of three independent experiments. Statistical analyses were performed using GraphPad Prism software. Differences between the two groups were analyzed using unpaired Student’s *t* tests, and comparisons among multiple groups were performed using one-way analysis of variance (ANOVA) followed by Tukey’s post hoc test. All *p* values were two-tailed, and *p* < 0.05 was considered statistically significant. ∗*p* < 0.05, ∗∗*p* < 0.01, and ∗∗∗*p* < 0.001.

## Data and code availability

The authors declare that all data are available within the article and supplemental information or can be obtained from the authors upon reasonable request.

## Acknowledgments

We thank all the participants for their generous support of the current study. This study was supported by the 10.13039/501100001809National Natural Science Foundation of China (82471150 and 82622029 to J.C., 82473310 and 82271149 to H. Wu, and 82674294 to Hongxiu Yu), the Noncommunicable Chronic Diseases-National Science and Technology Major Project (2023ZD0520200 to H. Wang), the 10.13039/501100013105Shanghai Rising-Star Program (23QA1400900 to J.C.), the Youth Medical Talents-Specialist Program (SHWSRS(2023)_062 to J.C.), the Shanghai Eastern Talent Program (SHSWRC(2023)_024 to J.C. and LJ2024111 to Hongxiu Yu), the Shanghai Clinical Research Center for Cell Therapy (23J41900100), the Medical Science Data Center in Shanghai Medical College of Fudan University, and the Core Facility of Shanghai Medical College of Fudan University. We thank the staff members of the Molecular Imaging System & Animal Facility (https://cstr.cn/31129.02.NFPS.MIS) at the National Facility for Protein Science in Shanghai (https://cstr.cn/31129.02.NFPS) for providing technical support and assistance with data collection and analysis.

## Author contributions

Conceptualization, J.C., H. Wang, and Hongxiu Yu; methodology, L.Z., X.X., Haiwen Yu, and S.Q.; investigation, L.Z., X.X., Haiwen Yu, S.Q., Z.W., Y.F., P.H., G.L., and Y.C.; software and bioinformatics, Z.W. and Haiwen Yu; validation, L.Z., X.X., Haiwen Yu, and S.Q.; writing – original draft, L.Z., X.X., Haiwen Yu, and S.Q.; writing – review & editing, J.C., H. Wu, H. Wang, Hongxiu Yu, L.C., L.Z., and X.X.; supervision, J.C., H. Wang, Hongxiu Yu, and L.C.; project administration, J.C., H. Wang, and L.C.; funding acquisition, J.C., Hongxiu Yu, H. Wang, and H. Wu. All authors reviewed and edited the manuscript.

## Declaration of interests

J.C., H. Wang, X.X., S.Q., and H. Wu are inventors on a pending Chinese patent application (202511191785X) related to this work.

## Declaration of generative AI and AI-assisted technologies in the manuscript preparation process

During the preparation of this work, the authors used ChatGPT to assist with language polishing. All content generated using this tool was carefully reviewed and edited by the authors, who take full responsibility for the final version of the manuscript.

## Supplemental information

Document S1. Figures S1–S7

Document S2. Article plus supplemental information

## References

[1] Cappell K.M., Kochenderfer J.N. (2023). Long-term outcomes following CAR T cell therapy: what we know so far. Nat. Rev. Clin. Oncol..

[2] Lee D.W., Kochenderfer J.N., Stetler-Stevenson M., Cui Y.K., Delbrook C., Feldman S.A., Fry T.J., Orentas R., Sabatino M., Shah N.N. (2015). T cells expressing CD19 chimeric antigen receptors for acute lymphoblastic leukaemia in children and young adults: a phase 1 dose-escalation trial. Lancet.

[3] Khalifeh M., Hopewell E., Salman H. (2025). CAR T cell therapy for treatment of Acute Myeloid Leukemia, Advances and Outcomes. Mol. Ther..

[4] Chen Y., Ren R., Yan L., Zhou Y., Sun R., Song H., Yan H., Li Y. (2025). From Bench to Bedside: Emerging Paradigms in CAR-T Cell Therapy for Solid Malignancies. Adv. Sci..

[5] Du B., Qin J., Lin B., Zhang J., Li D., Liu M. (2025). CAR-T therapy in solid tumors. Cancer Cell.

[6] Kwon N., Chen Y.Y. (2025). Overcoming solid-tumor barriers: armored CAR-T cell therapy. Trends Cancer.

[7] Khalifeh M., Salman H. (2025). Engineering resilient CAR T cells for immunosuppressive environment. Mol. Ther..

[8] Gao T.A., Shih R.M., Clubb J.D., Hung S.-H., Singh T., James-Allan L.B., DiBernardo G., Shafer A., Bouren A., Ayala Ceja M. (2026). Engineering CAR T cells to secrete VEGF-neutralizing scFvs enhances antitumor activity against solid tumors. Sci. Transl. Med..

[9] Nazha B., Inal C., Owonikoko T.K. (2020). Disialoganglioside GD2 Expression in Solid Tumors and Role as a Target for Cancer Therapy. Front. Oncol..

[10] Pellegatta S., Savoldo B., Di Ianni N., Corbetta C., Chen Y., Patané M., Sun C., Pollo B., Ferrone S., DiMeco F. (2018). Constitutive and TNFalpha-inducible expression of chondroitin sulfate proteoglycan 4 in glioblastoma and neurospheres: Implications for CAR-T cell therapy. Sci. Transl. Med..

[11] Morello A., Sadelain M., Adusumilli P.S. (2016). Mesothelin-Targeted CARs: Driving T Cells to Solid Tumors. Cancer Discov..

[12] Huang Y., Wang H. (2025). Tonic signaling in CAR-T therapy: the lever long enough to move the planet. Front. Med..

[13] Qi C., Liu D., Liu C., Wei X., Ma M., Lu X., Tao M., Zhang C., Wang X., He T. (2024). Antigen-independent activation is critical for the durable antitumor effect of GUCY2C-targeted CAR-T cells. J. Immunother. Cancer.

[14] Cappell K.M., Kochenderfer J.N. (2021). A comparison of chimeric antigen receptors containing CD28 versus 4-1BB costimulatory domains. Nat. Rev. Clin. Oncol..

[15] Long A.H., Haso W.M., Shern J.F., Wanhainen K.M., Murgai M., Ingaramo M., Smith J.P., Walker A.J., Kohler M.E., Venkateshwara V.R. (2015). 4-1BB costimulation ameliorates T cell exhaustion induced by tonic signaling of chimeric antigen receptors. Nat. Med..

[16] Shukla D., Gabunia K., McGettigan S.E., Patel P.R., Christensen S., Fan T.J., Song D., Luo Y., Wang Y., Wang H. (2025). CAR Binders Affect CAR T-cell Tonic Signaling, Durability, and Sensitivity to Target. Cancer Immunol. Res..

[17] Chen J., Qiu S., Li W., Wang K., Zhang Y., Yang H., Liu B., Li G., Li L., Chen M. (2023). Tuning charge density of chimeric antigen receptor optimizes tonic signaling and CAR-T cell fitness. Cell Res..

[18] Alabanza L., Pegues M., Geldres C., Shi V., Wiltzius J.J.W., Sievers S.A., Yang S., Kochenderfer J.N. (2017). Function of Novel Anti-CD19 Chimeric Antigen Receptors with Human Variable Regions Is Affected by Hinge and Transmembrane Domains. Mol. Ther..

[19] Watanabe N., Bajgain P., Sukumaran S., Ansari S., Heslop H.E., Rooney C.M., Brenner M.K., Leen A.M., Vera J.F. (2016). Fine-tuning the CAR spacer improves T-cell potency. Oncoimmunology.

[20] Petri K., D'Ippolito E., Künkele A., Köhl U., Busch D.H., Einsele H., Hudecek M. (2025). Next-generation T cell immunotherapies engineered with CRISPR base and prime editing: challenges and opportunities. Nat. Rev. Clin. Oncol..

[21] Knudsen N.H., Escobar G., Korell F., Kienka T., Nobrega C., Anderson S., Cheng A.Y., Zschummel M., Armstrong A., Bouffard A. (2025). In vivo CRISPR screens identify modifiers of CAR T cell function in myeloma. Nature.

[22] Datlinger P., Pankevich E.V., Arnold C.D., Pranckevicius N., Lin J., Romanovskaia D., Schaefer M., Piras F., Orts A.C., Nemc A. (2025). Systematic discovery of CRISPR-boosted CAR T cell immunotherapies. Nature.

[23] Barden M., Elsenbroich P.R., Haas V., Ertelt M., Pervan P., Velas L., Gergely B., Szöőr Á., Harrer D.C., Bezler V. (2024). Integrating binding affinity and tonic signaling enables a rational CAR design for augmented T cell function. J. Immunother. Cancer.

[24] Fink A., Sal-Man N., Gerber D., Shai Y. (2012). Transmembrane domains interactions within the membrane milieu: principles, advances and challenges. Biochim. Biophys. Acta.

[25] Muller Y.D., Nguyen D.P., Ferreira L.M.R., Ho P., Raffin C., Valencia R.V.B., Congrave-Wilson Z., Roth T.L., Eyquem J., Van Gool F. (2021). The CD28-Transmembrane Domain Mediates Chimeric Antigen Receptor Heterodimerization With CD28. Front. Immunol..

[26] Leddon S.A., Fettis M.M., Abramo K., Kelly R., Oleksyn D., Miller J. (2020). The CD28 Transmembrane Domain Contains an Essential Dimerization Motif. Front. Immunol..

[27] Philips E.A., Liu J., Kvalvaag A., Mørch A.M., Tocheva A.S., Ng C., Liang H., Ahearn I.M., Pan R., Luo C.C. (2024). Transmembrane domain-driven PD-1 dimers mediate T cell inhibition. Sci. Immunol..

[28] Zhang X., Schwartz J.-C.D., Almo S.C., Nathenson S.G. (2003). Crystal structure of the receptor-binding domain of human B7-2: Insights into organization and signaling. Proc. Natl. Acad. Sci. USA.

[29] Muhle-Goll C., Hoffmann S., Afonin S., Grage S.L., Polyansky A.A., Windisch D., Zeitler M., Bürck J., Ulrich A.S. (2012). Hydrophobic matching controls the tilt and stability of the dimeric platelet-derived growth factor receptor (PDGFR) β transmembrane segment. J. Biol. Chem..

[30] Zak K.M., Kitel R., Przetocka S., Golik P., Guzik K., Musielak B., Dömling A., Dubin G., Holak T.A. (2015). Structure of the Complex of Human Programmed Death 1, PD-1, and Its Ligand PD-L1. Structure.

[31] Xu X., Qiu S., Wang Z., Li D., Chen M., Chu Y., Li G., Fang Y., Li C., Shi F. (2026). Humanized and Charge-Optimized CSPG4-Specific CAR-T Cells show Enhanced Efficacy against Head and Neck Squamous Cell Carcinoma. Adv. Sci..

[32] Minegishi Y., Conley M.E. (2001). Negative selection at the pre-BCR checkpoint elicited by human mu heavy chains with unusual CDR3 regions. Immunity.

[33] Yamasaki S., Ishikawa E., Sakuma M., Ogata K., Sakata-Sogawa K., Hiroshima M., Wiest D.L., Tokunaga M., Saito T. (2006). Mechanistic basis of pre-T cell receptor-mediated autonomous signaling critical for thymocyte development. Nat. Immunol..

[34] Chen X., Chen L.C., Khericha M., Meng X., Salvestrini E., Shafer A., Iyer N., Alag A.S., Ding Y., Nicolaou D.M., Chen Y.Y. (2023). Rational Protein Design Yields a CD20 CAR with Superior Antitumor Efficacy Compared with CD19 CAR. Cancer Immunol. Res..

[35] Bernhardt N., Ozturk T.N., Zhang S., Schwartz N., Chadda R., Gil-Ley A., Robertson J.L., Faraldo-Gómez J.D. (2026). Molecular basis for the regulation of membrane proteins through preferential lipid solvation. Nat. Chem. Biol..

[36] Peruzzi J.A., Steinkühler J., Vu T.Q., Gunnels T.F., Hu V.T., Lu P., Baker D., Kamat N.P. (2024). Hydrophobic mismatch drives self-organization of designer proteins into synthetic membranes. Nat. Commun..

[37] Kim T., Im W. (2010). Revisiting hydrophobic mismatch with free energy simulation studies of transmembrane helix tilt and rotation. Biophys. J..

[38] Guerrero J.A., Klysz D.D., Chen Y., Malipatlolla M., Lone J., Fowler C., Stuani L., May A., Bashti M., Xu P. (2024). GLUT1 overexpression in CAR-T cells induces metabolic reprogramming and enhances potency. Nat. Commun..

[39] Weber E.W., Parker K.R., Sotillo E., Lynn R.C., Anbunathan H., Lattin J., Good Z., Belk J.A., Daniel B., Klysz D. (2021). Transient rest restores functionality in exhausted CAR-T cells through epigenetic remodeling. Science.

[40] Lynn R.C., Weber E.W., Sotillo E., Gennert D., Xu P., Good Z., Anbunathan H., Lattin J., Jones R., Tieu V. (2019). c-Jun overexpression in CAR T cells induces exhaustion resistance. Nature.

[41] Gattinoni L., Lugli E., Ji Y., Pos Z., Paulos C.M., Quigley M.F., Almeida J.R., Gostick E., Yu Z., Carpenito C. (2011). A human memory T cell subset with stem cell-like properties. Nat. Med..

[42] Shi Y., Lopez M.A., Kotchetkov I.S., Jain N., Zhao Z., Halim L., Dobrin A., Perica K., Hanina S.A., Rajasekhar V.K. (2026). pTα enhances mRNA translation and potentiates CAR T cells for solid tumor eradication. Cell.

[43] Frischholz S., Schuster E.M., Grotz M., Schülein C., Benz J., Kocher K., Klotz L., Varga S., Hiltner T., Alsalameh R. (2026). Metabolic quiescence of naive-like memory T cells precedes and maintains antigen-specific T cell memory. Nat. Immunol..

[44] Choi S., Matta H., Gopalakrishnan R., Natarajan V., Gong S., Jeronimo A., Kuo W.Y., Bravo B., Chaudhary P.M. (2021). A novel thermostable beetle luciferase based cytotoxicity assay. Sci. Rep..

[45] Velasco Cárdenas R.M.H., Brandl S.M., Meléndez A.V., Schlaak A.E., Buschky A., Peters T., Beier F., Serrels B., Taromi S., Raute K. (2023). Harnessing CD3 diversity to optimize CAR T cells. Nat. Immunol..

[46] Sommermeyer D., Hudecek M., Kosasih P.L., Gogishvili T., Maloney D.G., Turtle C.J., Riddell S.R. (2016). Chimeric antigen receptor-modified T cells derived from defined CD8+ and CD4+ subsets confer superior antitumor reactivity in vivo. Leukemia.

[47] Landoni E., Fucá G., Wang J., Chirasani V.R., Yao Z., Dukhovlinova E., Ferrone S., Savoldo B., Hong L.K., Shou P. (2021). Modifications to the Framework Regions Eliminate Chimeric Antigen Receptor Tonic Signaling. Cancer Immunol. Res..

[48] Bartleson J.M., Viehmann Milam A.A., Donermeyer D.L., Horvath S., Xia Y., Egawa T., Allen P.M. (2020). Strength of tonic T cell receptor signaling instructs T follicular helper cell–fate decisions. Nat. Immunol..

[49] Pang S.S., Berry R., Chen Z., Kjer-Nielsen L., Perugini M.A., King G.F., Wang C., Chew S.H., La Gruta N.L., Williams N.K. (2010). The structural basis for autonomous dimerization of the pre-T-cell antigen receptor. Nature.

[50] Wang X., Tao X., Chen P., Jiang P., Li W., Chang H., Wei C., Lai X., Zhang H., Pan Y. (2024). MEK inhibition prevents CAR-T cell exhaustion and differentiation via downregulation of c-Fos and JunB. Signal Transduct. Targeted Ther..

[51] Tang J., Sheng J., Zhang Q., Ji Y., Wang X., Zhang J., Wu J., Song J., Bai X., Liang T. (2023). Runx3-overexpression cooperates with ex vivo AKT inhibition to generate receptor-engineered T cells with better persistence, tumor-residency, and antitumor ability. J. Immunother. Cancer.

[52] Wang H., Huang Y., Xu C. (2023). Charging CAR by electrostatic power. Immunol. Rev..

[53] Wang H., Song X., Shen L., Wang X., Xu C. (2022). Exploiting T cell signaling to optimize engineered T cell therapies. Trends Cancer.

[54] Li W., Qiu S., Chen J., Jiang S., Chen W., Jiang J., Wang F., Si W., Shu Y., Wei P. (2020). Chimeric Antigen Receptor Designed to Prevent Ubiquitination and Downregulation Showed Durable Antitumor Efficacy. Immunity.

[55] Labanieh L., Majzner R.G., Klysz D., Sotillo E., Fisher C.J., Vilches-Moure J.G., Pacheco K.Z.B., Malipatlolla M., Xu P., Hui J.H. (2022). Enhanced safety and efficacy of protease-regulated CAR-T cell receptors. Cell.

[56] Freitas K.A., Belk J.A., Sotillo E., Quinn P.J., Ramello M.C., Malipatlolla M., Daniel B., Sandor K., Klysz D., Bjelajac J. (2022). Enhanced T cell effector activity by targeting the Mediator kinase module. Science.

[57] Maeta S., Fukunaga A., Egashira Y., Akahori Y., Wang L., Seo N., Atarashi Y., Ejima D., Fujiwara H., Shiku H. (2026). Introducing arginine residues into scFv in CAR-T cells shows promising antitumor effects. Mol. Ther..

[58] DeGolier K.R., Pham-Danis C., Burciaga S.D., Walsh Z.H., Brzezinski C., Novak A.J., Leach L., Cimons J., Li W., Zhongyu Z. (2026). Rational redesign of antigen binding domain improves in vivo efficacy of the CD22-CAR. Mol. Ther..

[59] Wang P., Wang Y., Zhao X., Zheng R., Zhang Y., Meng R., Dong H., Liang S., He X., Song Y. (2025). Chimeric antigen receptor with novel intracellular modules improves antitumor performance of T cells. Signal Transduct. Targeted Ther..

[60] Chang T.C., Heard A., Lattin J., Warrington J.M., Barrett A., Landmann J.H., Tenzin Y., Ganesh V., Thompson B., Afrin S. (2026). BACH2 regulates T cell lineage state to enhance CAR T cell function. Nat. Immunol..

[61] Huang Y., Zhang Q., Wang K., Ren X., He H., Wang J., Li L., Tan Z., Liu J., Zhu G., Wang H. (2026). FDA-approved fulvestrant-induced CAR phase separation enables precise control of CAR T antitumor function. Cell Stem Cell.

[62] Bjelajac J.R., Cañellas-Socias A., Nehra P., Reynolds K., Malipatlolla M., Martinez Velez N., Manjarrez D.C., Ho K., Xu P., Hamad J.L. (2026). Cell-autonomous control of CAR signaling and receptor shedding via ADAM17-mediated proteolysis. Cell.

[63] Singh N., Frey N.V., Engels B., Barrett D.M., Shestova O., Ravikumar P., Cummins K.D., Lee Y.G., Pajarillo R., Chun I. (2021). Antigen-independent activation enhances the efficacy of 4-1BB-costimulated CD22 CAR T cells. Nat. Med..

[64] Sánchez M.F., Tampé R. (2023). Ligand-independent receptor clustering modulates transmembrane signaling: a new paradigm. Trends Biochem. Sci..

[65] Wu H., Cao R., Wen M., Xue H., OuYang B. (2022). Structural characterization of a dimerization interface in the CD28 transmembrane domain. Structure.

[66] Lamarche C., Ward-Hartstonge K., Mi T., Lin D.T.S., Huang Q., Brown A., Edwards K., Novakovsky G.E., Qi C.N., Kobor M.S. (2023). Tonic-signaling chimeric antigen receptors drive human regulatory T cell exhaustion. Proc. Natl. Acad. Sci. USA.

[67] Wang H., Wu Z., Cui D., Bian L., Zheng Z., Zhu J., Geng H., Sun Z., Pan Y., Shi Y. (2025). Triple knockdown of CD11a, CD49d, and PSGL1 in T cells reduces CAR-T cell toxicity but preserves activity against solid tumors in mice. Sci. Transl. Med..

[68] Klysz D.D., Fowler C., Malipatlolla M., Stuani L., Freitas K.A., Chen Y., Meier S., Daniel B., Sandor K., Xu P. (2024). Inosine induces stemness features in CAR-T cells and enhances potency. Cancer Cell.

[69] Isshiki Y., Chen X., Teater M., Karagiannidis I., Nam H., Cai W., Meydan C., Xia M., Shen H., Gutierrez J. (2025). EZH2 inhibition enhances T cell immunotherapies by inducing lymphoma immunogenicity and improving T cell function. Cancer Cell.

[70] Eyquem J., Mansilla-Soto J., Giavridis T., van der Stegen S.J.C., Hamieh M., Cunanan K.M., Odak A., Gönen M., Sadelain M. (2017). Targeting a CAR to the TRAC locus with CRISPR/Cas9 enhances tumour rejection. Nature.

[71] Ahn T., Bae E.-A., Seo H. (2024). Decoding and overcoming T cell exhaustion: Epigenetic and transcriptional dynamics in CAR-T cells against solid tumors. Mol. Ther..

[72] Zhu Z., Luo Y., Lou G., Yihunie K., Wizzard S., DeVilbiss A.W., Muh S., Ma C., Shinde S.S., Hoar J. (2024). The redox sensor KEAP1 facilitates adaptation of T cells to chronic antigen stimulation by preventing hyperactivation. Sci. Immunol..

[73] Zhu Q., Li J., Liu N., Han L., Wu Z., Wang Y., Lin X., Wei J., Han W. (2025). CD2 augmentation enhances CAR-T-cell efficacy via immunological synapse remodeling and T-cell exhaustion mitigation. Cell. Mol. Immunol..

[74] Xu X., Chen H., Ren Z., Xu X., Wu W., Yang H., Wang J., Zhang Y., Zhou Q., Li H. (2024). Phase separation of chimeric antigen receptor promotes immunological synapse maturation and persistent cytotoxicity. Immunity.

[75] Canning A., Donohoe E., Johnston É., Lynch K., Moosavizadeh S., Wang J., Leahy M., Treacy O., Ryan A.E., Ritter T. (2026). Subconjunctival administration of allogeneic TNF-α/IL-1β licensed MSCs promotes an anti-inflammatory microenvironment in a murine model of severe alkali burn. Int. Immunopharmacol..

[76] Lynch K., Treacy O., Chen X., Murphy N., Lohan P., Islam M.N., Donohoe E., Griffin M.D., Watson L., McLoughlin S. (2020). TGF-β1-Licensed Murine MSCs Show Superior Therapeutic Efficacy in Modulating Corneal Allograft Immune Rejection In Vivo. Mol. Ther..

[77] Arcangeli S., Bove C., Mezzanotte C., Camisa B., Falcone L., Manfredi F., Bezzecchi E., El Khoury R., Norata R., Sanvito F. (2022). CAR T cell manufacturing from naive/stem memory T lymphocytes enhances antitumor responses while curtailing cytokine release syndrome. J. Clin. Investig..

[78] Schultz L.M., Czerwinski D.K., Levy R., Levy S. (2022). CD81 costimulation skews CAR transduction toward naive T cells. Proc. Natl. Acad. Sci. USA.

[79] Carrillo M.A., Zhen A., Mu W., Rezek V., Martin H., Peterson C.W., Kiem H.P., Kitchen S.G. (2024). Stem cell-derived CAR T cells show greater persistence, trafficking, and viral control compared to ex vivo transduced CAR T cells. Mol. Ther..

[80] Hallgren J., Tsirigos K.D., Pedersen M.D., Almagro Armenteros J.J., Marcatili P., Nielsen H., Krogh A., Winther O. (2022). DeepTMHMM predicts alpha and beta transmembrane proteins using deep neural networks. bioRxiv.

[81] Waterhouse A., Bertoni M., Bienert S., Studer G., Tauriello G., Gumienny R., Heer F.T., de Beer T.A.P., Rempfer C., Bordoli L. (2018). SWISS-MODEL: homology modelling of protein structures and complexes. Nucleic Acids Res..

[82] Lomize A.L., Todd S.C., Pogozheva I.D. (2022). Spatial arrangement of proteins in planar and curved membranes by PPM 3.0. Protein Sci..

