# Supplementary material for: PD-L1 transmembrane domain substitution overcomes CAR-T exhaustion driven by tonic signaling
Source: Mol Ther Oncol. 2026 Oct 14;34(4):201364. doi: 10.1016/j.omton.2026.201364 (PMC13742466; doi:10.1016/j.omton.2026.201364)
Supplement: Supplementary file 1 [file mmc1.pdf]

OMTON, Volume *34*

Supplemental information

PD-L1 transmembrane domain substitution overcomes CAR-T exhaustion driven by tonic signaling

Liang Zhang, Xiang Xu, Haiwen Yu, Shizhen Qiu, Zhitong Wang, Yinying Chu, Guangfei Li, Yi Fang, Peijie He, Haitao Wu, Lei Cheng, Hongxiu Yu, Haopeng Wang, and Jian Chen

Figure S1

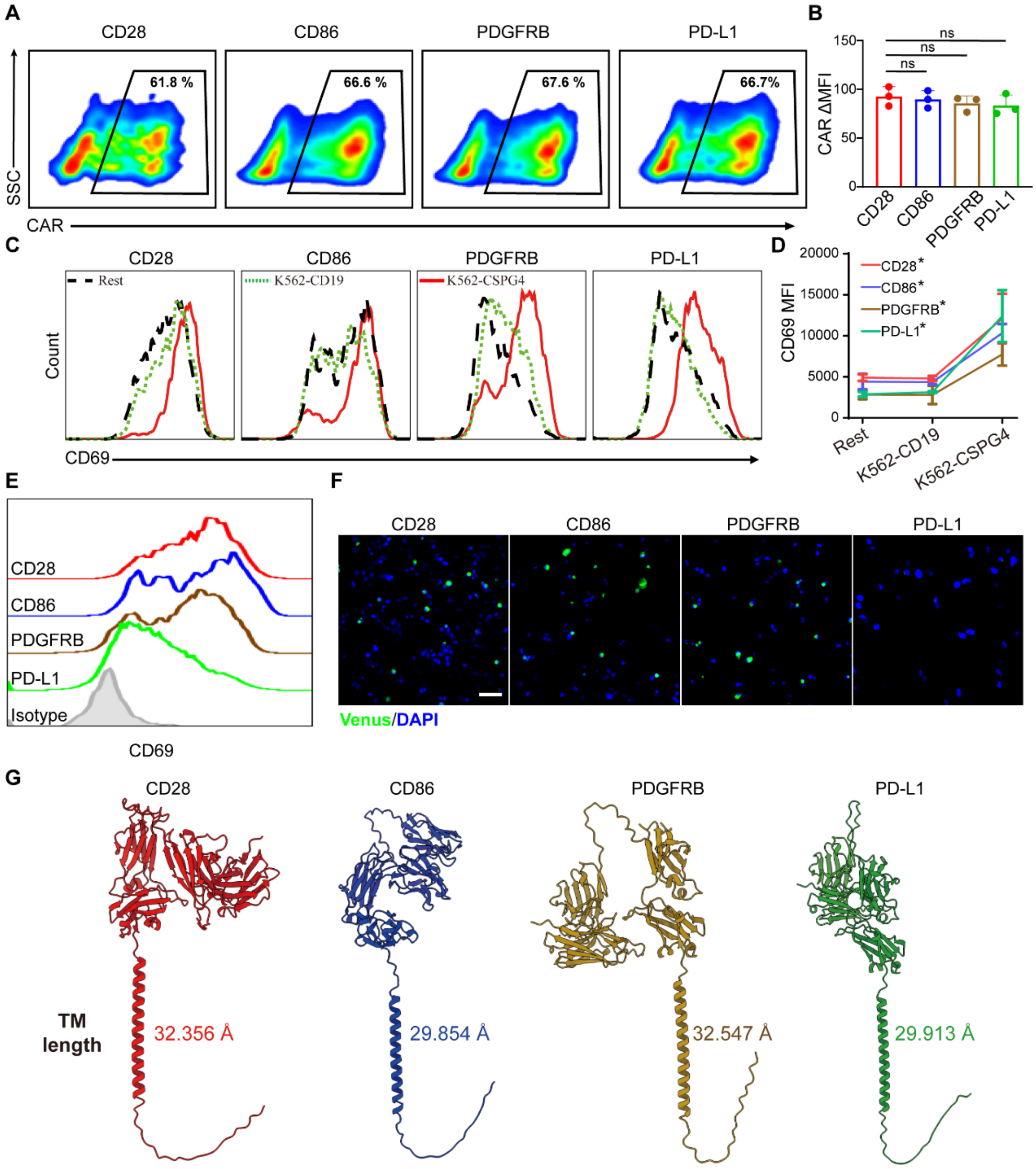
A
CD28
CD86
PDGFRB
PD-L1
61.8 %
66.6 %
67.6 %
66.7%
SSC
CAR
B
ns
ns
ns
CAR ΔMFI
150
100
50
0
CD28
CD86
PDGFRB
PD-L1
C
CD28
CD86
PDGFRB
PD-L1
Rest
K562-CD19
K562-CSPG4
Count
CD69
D
20000
15000
10000
5000
0
CD69 MFI
CD28*
CD86*
PDGFRB*
PD-L1*
Rest
K562-CD19
K562-CSPG4
E
CD28
CD86
PDGFRB
PD-L1
Isotype
CD69
F
CD28
CD86
PDGFRB
PD-L1
Venus/DAPI
G
CD28
CD86
PDGFRB
PD-L1
TM length
32.356 Å
29.854 Å
32.547 Å
29.913 Å

Figure S1. Characterization of CAR expression, antigen specificity, and structural modeling in Jurkat cells (related to Figure 1).

(A) Representative flow cytometry plots showing CAR-positive percentage on Jurkat cells 72 hours after transduction with the indicated CSPG4 CAR constructs. CAR expression was detected using a fluorochrome-conjugated anti-hIgG antibody.

(B) Quantification of CAR expression levels, measured as relative MFI by flow cytometry. Data represent mean ± SEM of n = 3 biological independent experiments. n.s., not significant; unpaired Student's t-test relative to CD28 TM.

(C) Representative flow cytometry histograms of CD69 expression on CAR-Jurkat cells under three conditions: resting (black), after 24 hours co-culture with non-target K562-CD19 cells (green), or with target K562-CSPG4 cells (red) at an effector-to-target ratio of 1:1.

(D) Quantification of antigen-specific CD69 upregulation. The fold change in CD69 MFI upon co-culture with target K562-CSPG4 cells versus non-target K562-CD19 cells is shown. Data represent mean ± SEM of n = 3 biological independent experiments. *P < 0.05, **P < 0.01, ***P < 0.001; paired two-tailed t-test (target vs. non-target).

(E) Representative flow cytometry histograms of CD69 expression on CAR-Jurkat cells under resting conditions, illustrating the gradient of tonic signaling across the four CAR variants.

(F) Representative live-cell fluorescence microscopy images of the BiFC assay. Venus fluorescence (green) indicates CAR-CAR self-association in intact, living Jurkat cells. Nuclei are counterstained with Hoechst (blue). Scale bar, 50 μm.

(G) Predicted 3D structural models of the TM domains for each CAR construct. The calculated lengths of the transmembrane helical regions are indicated.

Figure S2

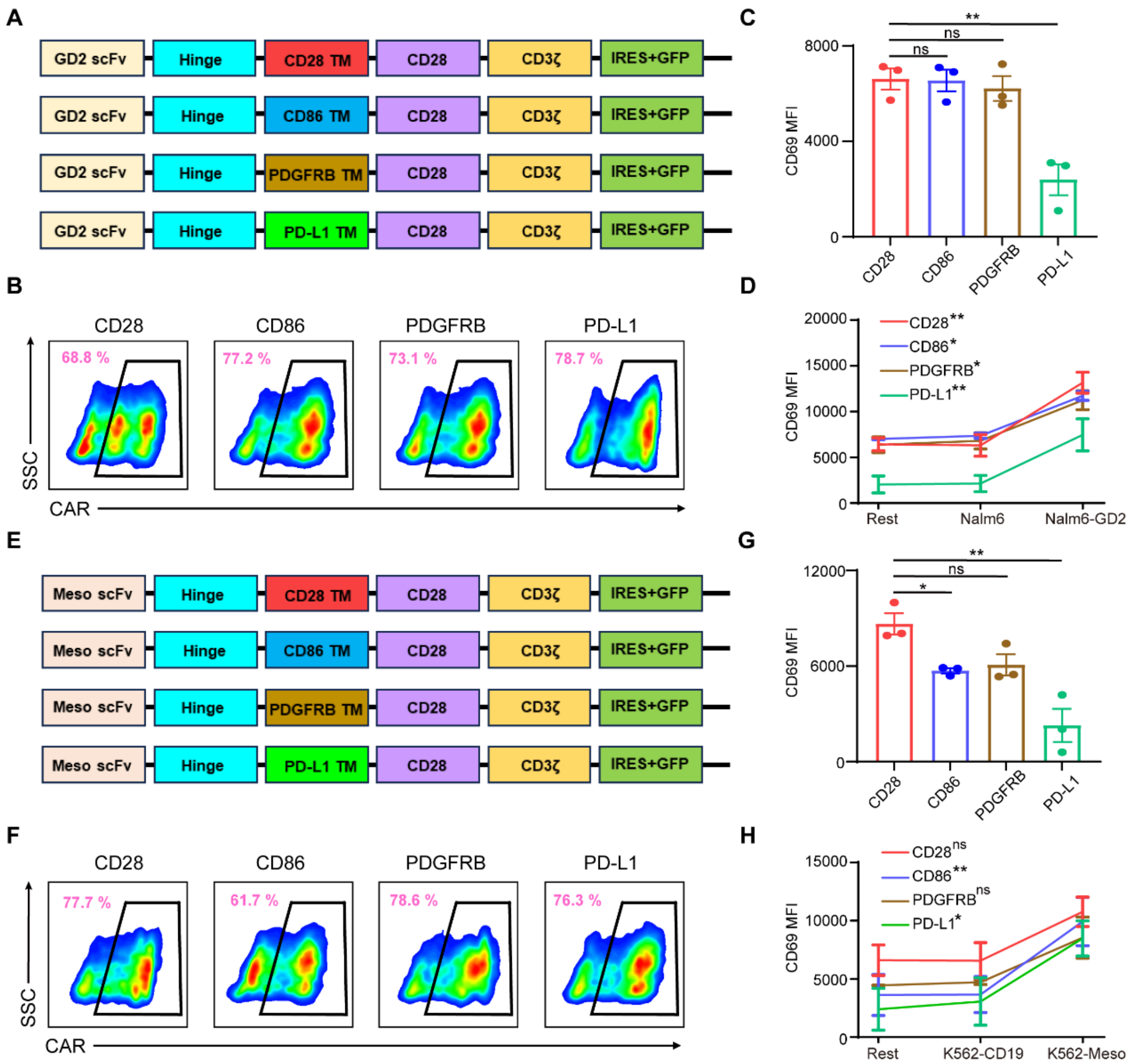
A
GD2 scFv
Hinge
CD28 TM
CD86 TM
PDGFRB TM
PD-L1 TM
CD28
CD3ζ
IRES+GFP
B
CD28
CD86
PDGFRB
PD-L1
68.8 %
77.2 %
73.1 %
78.7 %
SSC
CAR
C
CD69 MFI
**
ns
ns
D
Rest
Nalm6
Nalm6-GD2
CD28**
CD86*
PDGFRB*
PD-L1**
E
Meso scFv
F
77.7 %
61.7 %
78.6 %
76.3 %
G
*
H
K562-CD19
K562-Meso
CD28ns
CD86**
PDGFRBns
PD-L1*

Figure S2. The PD-L1 transmembrane domain reduces tonic signaling across multiple CAR specificities (related to Figure 1).

(A) Schematic representation of GD2-targeted CAR constructs.

(B) Representative flow cytometry plots showing CAR-positive percentage on Jurkat cells 72 hours after transduction with the indicated GD2 CAR constructs. CAR expression was detected using a fluorochrome-conjugated anti-hIgG antibody.

(C) Quantification of tonic signaling, measured as CD69 MFI in CAR-transduced Jurkat cells cultured in the absence of antigen. Data represent mean ± SEM of n = 3 biological independent experiments. P values were determined by unpaired Student's t-test relative to CD28 TM. **P < 0.01; n.s., not significant.

(D) Quantification of antigen-specific CD69 upregulation. The fold change in CD69 MFI upon co-culture with target Nalm6-GD2 cells versus non-target Nalm6 cells is shown. Data represent mean ± SEM of n = 3 biological independent experiments. *P < 0.05, **P < 0.01; paired two-tailed t-test (target vs. non-target).

(E) Schematic representation of mesothelin (Meso)-targeted CAR constructs.

(F) Representative flow cytometry plots showing CAR-positive percentage on Jurkat cells 72 hours after transduction with the indicated GD2 CAR constructs. CAR expression was detected using a fluorochrome-conjugated anti-myc-tag antibody.

(G) Quantification of tonic signaling, measured as CD69 MFI in CAR-transduced Jurkat cells cultured in the absence of antigen. Data represent mean ± SEM of n = 3 biological independent experiments. P values were determined by unpaired Student's t-test relative to CD28 TM. **P < 0.01; n.s., not significant.

(H) Quantification of antigen-specific CD69 upregulation in Meso CAR-Jurkat cells after 24 hours co-culture with non-target K562-CD19 cells or target K562-Meso cells at an E:T ratio of 1:1. The fold change in CD69 MFI (target vs. non-target) is shown. Data represent mean ± SEM of n = 3 biological independent experiments. *P < 0.05, **P < 0.01; paired two-tailed t-test (target vs. non-target).

Figure S3

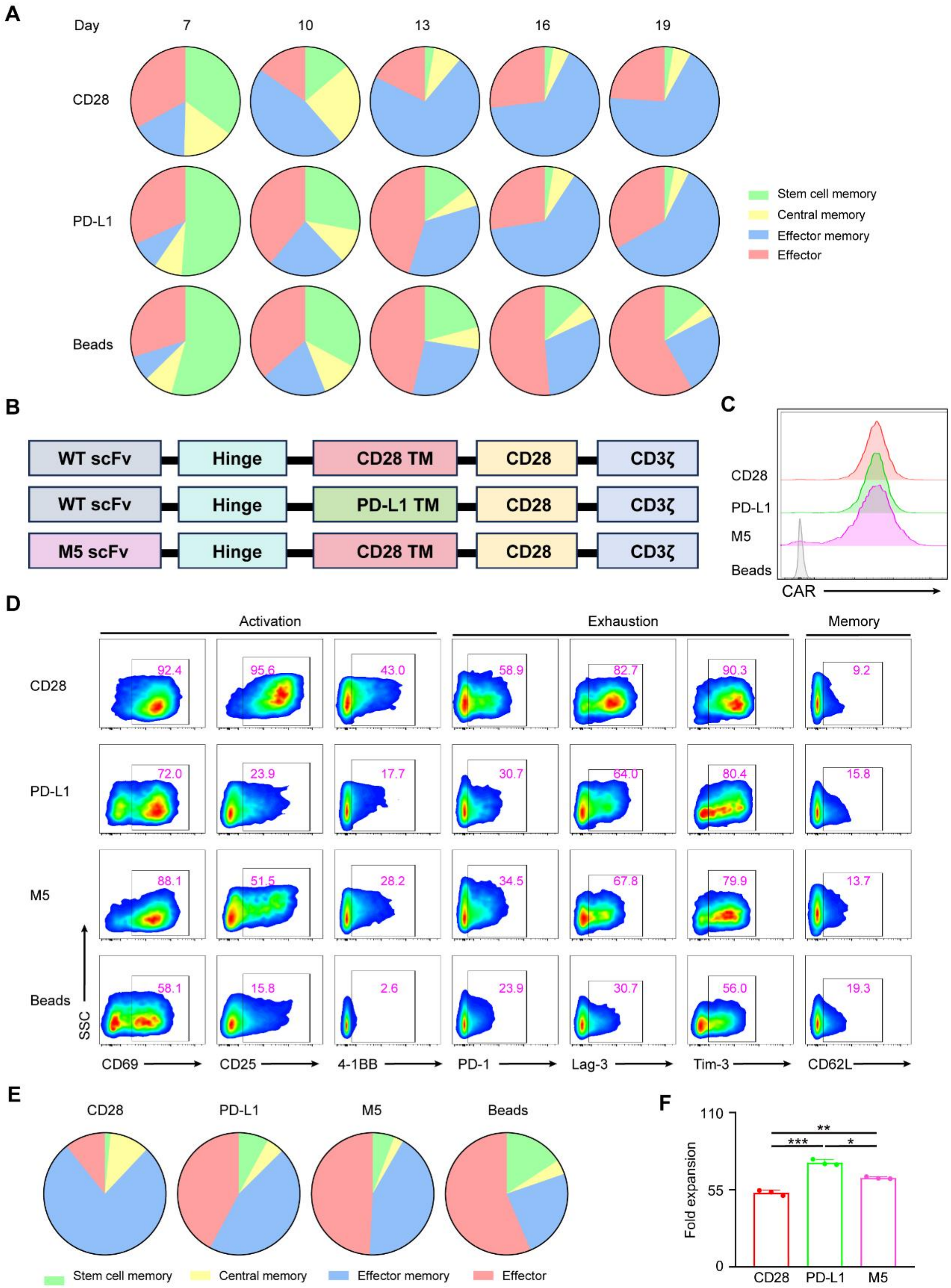
A
Day
7
10
13
16
19
CD28
PD-L1
Beads
Stem cell memory
Central memory
Effector memory
Effector
B
WT scFv
Hinge
CD28 TM
CD28
CD3ζ
WT scFv
Hinge
PD-L1 TM
CD28
CD3ζ
M5 scFv
Hinge
CD28 TM
CD28
CD3ζ
C
CD28
PD-L1
M5
Beads
CAR
D
Activation
Exhaustion
Memory
CD28
92.4
95.6
43.0
58.9
82.7
90.3
9.2
PD-L1
72.0
23.9
17.7
30.7
64.0
80.4
15.8
M5
88.1
51.5
28.2
34.5
67.8
79.9
13.7
Beads
58.1
15.8
2.6
23.9
30.7
56.0
19.3
SSC
CD69
CD25
4-1BB
PD-1
Lag-3
Tim-3
CD62L
E
CD28
PD-L1
M5
Beads
Stem cell memory
Central memory
Effector memory
Effector
F
110
55
0
Fold expansion
**
***
*
CD28
PD-L1
M5

Figure S3. Comparison of PD-L1 TM and scFv-optimized M5 CSPG4 CAR-T cells (related to Figure 2).

(A) Longitudinal quantification of T cell differentiation subsets from day 7 to day 19 in CD28 TM, PD-L1 TM, and Beads groups. Data represent mean ± SEM. Tscm, $CD45RA^{+}CD62L^{+}$; Tcm, $CD45RA^{-}CD62L^{+}$; Tem, $CD45RA^{-}CD62L^{-}$; Teff, $CD45RA^{+}CD62L^{-}$.

(B) Schematic of M5 (scFv-optimized), WT (CD28 TM), and PD-L1 TM CAR constructs. Briefly, human T lymphocytes were activated with CD3/CD28 Dynabeads on day 0 and transduced 48 hours later with lentivirus expressing the indicated second-generation CSPG4 CAR. At day 14 post-transduction, CAR surface expressions were detected using an anti-human IgG antibody, and CAR-T cell phenotypes were assessed by flow cytometry.

(C) CAR surface expressions of the three constructs in (B) were detected by anti-hIgG antibody.

(D) Tonic signaling (CD69, CD25, 4-1BB), exhaustion (PD-1, Lag-3, Tim-3), and memory (CD62L) markers in the three CAR-T cell groups.

(E) Memory subset distribution in the three CAR-T cell groups.

(F) Cumulative fold expansion of the indicated CAR-T cell groups over 14 days of culture. Data represent mean ± SEM of n = 3 biological independent experiments, each performed with a different donor. *$P < 0.05$, **$P < 0.01$, ***$P < 0.001$.

Figure S4

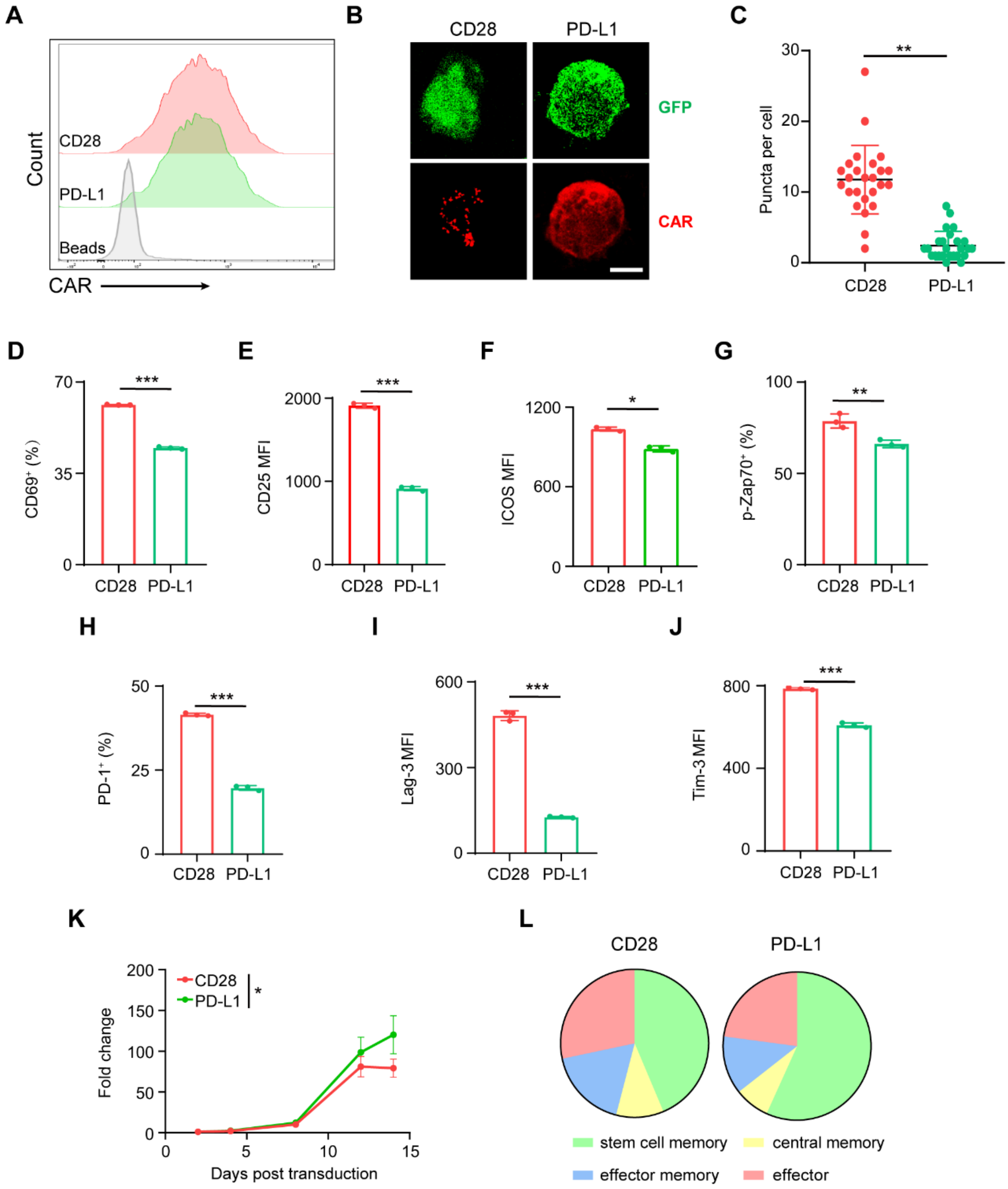
A
Count
CD28
PD-L1
Beads
CAR
B
CD28
PD-L1
GFP
CAR
C
Puncta per cell
**
CD28
PD-L1
D
CD69⁺ (%)
***
E
CD25 MFI
***
F
ICOS MFI
*
G
p-Zap70⁺ (%)
**
H
PD-1⁺ (%)
***
I
Lag-3 MFI
***
J
Tim-3 MFI
***
K
Fold change
CD28
PD-L1
*
Days post transduction
L
CD28
PD-L1
stem cell memory
central memory
effector memory
effector

Figure S4. The PD-L1 TM reduces tonic signaling and exhaustion in GD2 CAR-T cells (related to Figure 2).

(A) Human T lymphocytes were activated with CD3/CD28 Dynabeads and transduced 48 hours later with lentivirus expressing the indicated second-generation GD2 CAR. At day 14 post-transduction, CAR surface expressions were detected by anti-human IgG staining and CAR-T cell phenotypes were analyzed by flow cytometry. Representative flow cytometry plots showing GD2 CAR expression on T cells 14 days post-transduction.

(B) Representative immunofluorescence images of CAR clustering on Jurkat cells expressing the indicated GD2 CARs. Red, CAR; green, EGFP. Scale bars, 5 μm.

(C) Quantification of CAR puncta per cell shown in (B). Data represent mean ± SEM of n = 20 cells per group.

(D–F) Flow cytometric quantification of CD69 (D), CD25 (E), and ICOS (F) expression on GD2 CAR-T cells 14 days post-transduction. Data represent mean ± SEM of n = 3 biological independent experiments, each performed with a different donor.

(G) Flow cytometric quantification of phospho-ZAP70 expression on GD2 CAR-T cells 14 days post-transduction. Data represent mean ± SEM of n = 3 biological independent experiments.

(H–J) Flow cytometric quantification of PD-1 (H), Lag-3 (I), and Tim-3 (J) expression on GD2 CAR-T cells 14 days post-transduction. Data represent mean ± SEM of n = 3 biological independent experiments, each performed with a different donor.

(K) Cumulative fold expansion of GD2 CAR-T cells during ex vivo culture. Data represent mean ± SEM of n = 3 biological independent experiments, each performed with a different donor.

(L) Memory subset distribution of GD2 CAR-T cells 14 days post-transduction. Tscm, $CD45RA^{+}CD62L^{+}$; Tcm, $CD45RA^{-}CD62L^{+}$; Tem, $CD45RA^{-}CD62L^{-}$; Teff, $CD45RA^{+}CD62L^{-}$.

Figure S5

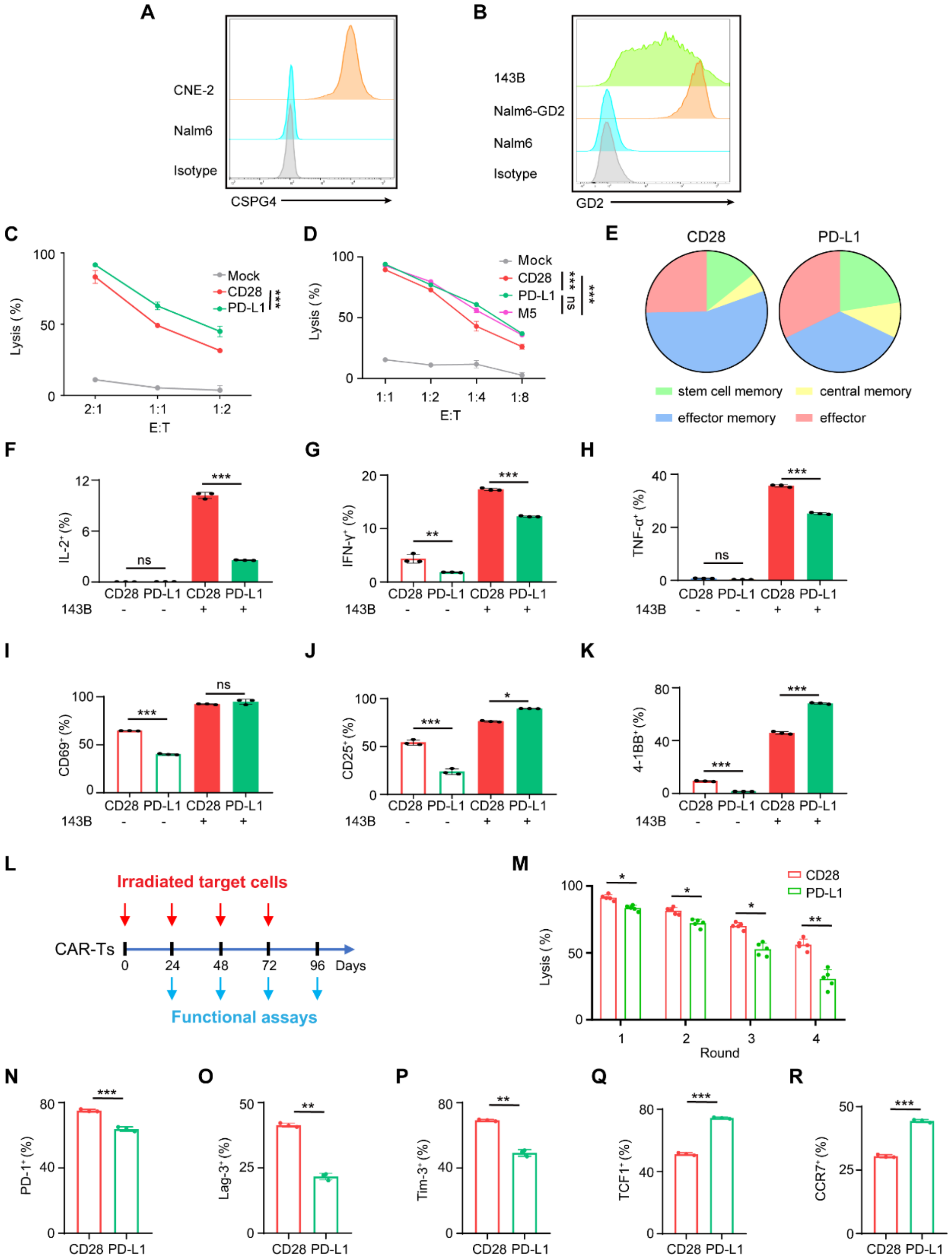

A
CNE-2
Nalm6
Isotype
CSPG4
B
143B
Nalm6-GD2
Nalm6
Isotype
GD2
C
Lysis (%)
Mock
CD28
PD-L1
***
E:T
D
Mock
CD28
PD-L1
M5
***
ns
***
E:T
E
CD28
PD-L1
stem cell memory
central memory
effector memory
effector
F
IL-2+ (%)
G
IFN-γ+ (%)
H
TNF-α+ (%)
I
CD69+ (%)
J
CD25+ (%)
K
4-1BB+ (%)
143B
L
Irradiated target cells
CAR-Ts
0
24
48
72
96
Days
Functional assays
M
CD28
PD-L1
Lysis (%)
Round
N
PD-1+ (%)
O
Lag-3+ (%)
P
Tim-3+ (%)
Q
TCF1+ (%)
R
CCR7+ (%)

Figure S5. PD-L1 TM GD2 CAR-T cells exhibit enhanced and sustained tumor cell killing (related to Figure 3).

(A) Flow cytometric validation of CSPG4 surface expression on CNE-2 cells.

(B) Flow cytometric validation of GD2 surface expression on Nalm6-GD2 and 143B cells.

(C) GD2 CAR-T cell cytotoxicity against live Nalm6-GD2 cells was measured by FFLuc reporter assay at the indicated E:T ratios 14 days after lentiviral transduction. Data represent mean ± SEM of n = 3 biological independent experiments, each performed with a different donor.

(D) Cytotoxicity of M5, CD28 TM, and PD-L1 TM CSPG4 CAR-T cells against CNE-2 cells was compared 14 days after lentiviral transduction. Data represent mean ± SEM of n = 3 biological independent experiments, each performed with a different donor.

(E) Memory phenotype of GD2 CAR-T cells was assessed 24 hours after co-culture with live Nalm6-GD2 cells at 14 days post-transduction. Tscm, $CD45RA^{+}CD62L^{+}$; Tcm, $CD45RA^{-}CD62L^{+}$; Tem, $CD45RA^{-}CD62L^{-}$; Teff, $CD45RA^{+}CD62L^{-}$.

(F-H) Fourteen days after transduction, GD2 CAR-T cells were stimulated with 143B cells for 8 hours, and intracellular cytokine staining for IL-2 (F), IFN-γ (G), and TNF-α (H) was performed before and after stimulation. Data represent mean ± SEM of n = 3 biological independent experiments, each performed with a different donor.

(I-K) Fourteen days after lentiviral transduction, surface expression of CD69 (I), CD25 (J), and 4-1BB (K) on GD2 CAR-T cells was analyzed before and after 24 hours stimulation with 143B cells. Data represent mean ± SEM of n = 3 biological independent experiments, each performed with a different donor.

(L) Schematic overview of the repetitive antigen stimulation protocol. CAR-T cells were restimulated with irradiated target cells at a 5:1 E:T ratio for four rounds, beginning on day 7 post-transduction and ending on day 12.

(M) Cytolytic activity of GD2 CAR-T cells against live Nalm6-GD2 cells after the 1st to 4th rounds of stimulation with irradiated Nalm6-GD2 cells, assessed by a 24 hours FFLuc reporter assay at a 1:1 E:T ratio. Data represent mean ± SEM of n = 4 biological independent experiments, each performed with a different donor.

(N-P) Expression of PD-1 (N), Lag-3 (O), and Tim-3 (P) on GD2 CAR-T cells after the fourth stimulation. Data represent mean ± SEM of n = 3 biological independent experiments, each performed with a different donor.

(Q-R) Flow cytometric quantification of $TCF1^{+}$ (Q) and $CCR7^{+}$ (R) frequencies in GD2 CAR-T cells after the fourth stimulation. Data represent mean ± SEM of n = 3 biological independent experiments, each performed with a different donor.

Figure S6

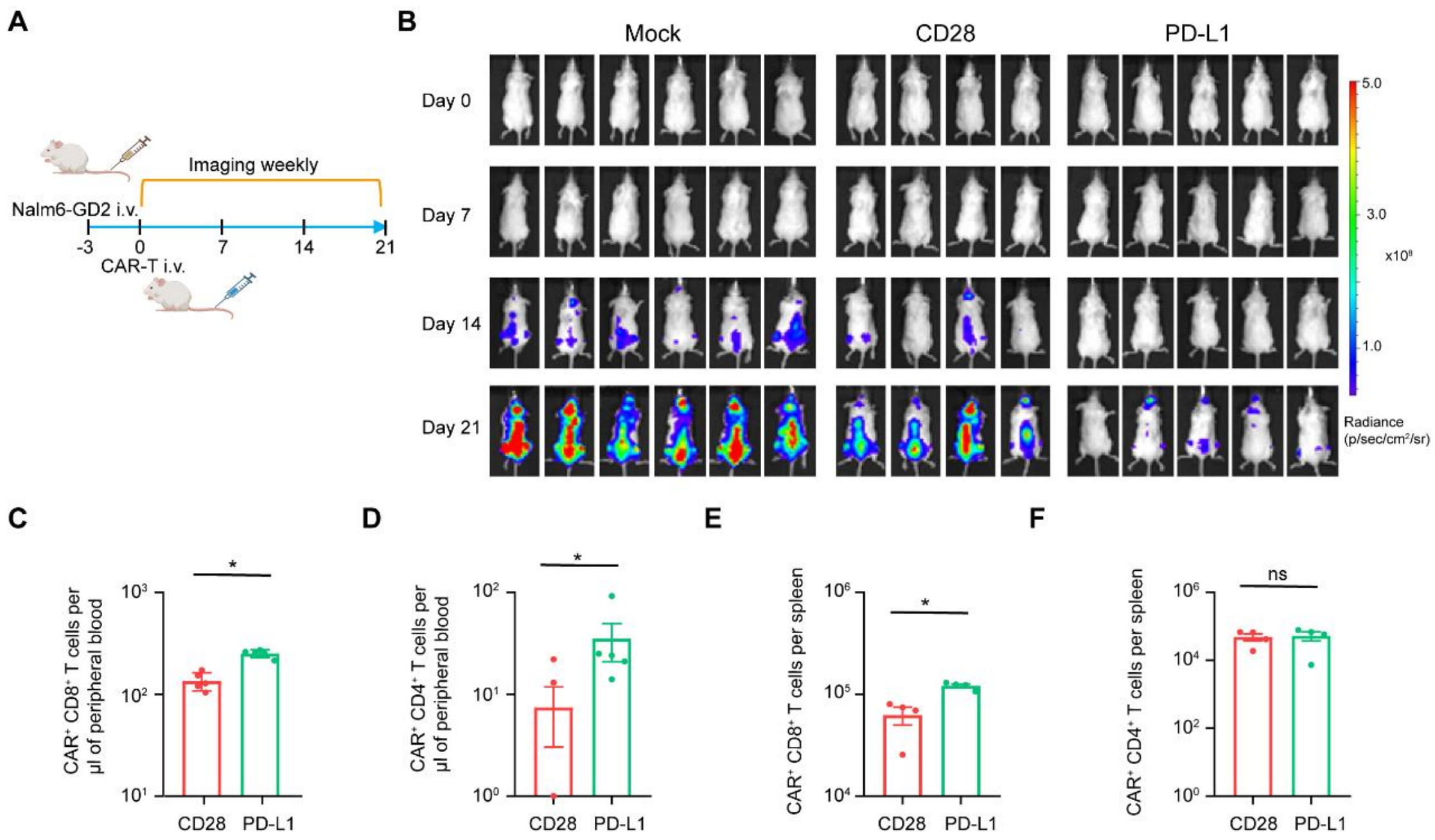

Figure S6. PD-L1 TM GD2 CAR-T cells show enhanced antitumor activity and persistence in a disseminated Nalm6-GD2 model (related to Figure 4).

(A) Schematic of the experimental timeline.

(B) Representative bioluminescence images and quantification of total flux.

(C-D) Absolute counts of CAR$^+$CD8$^+$ (C) and CAR$^+$ CD4$^+$ (D) T cells per µl peripheral blood on day 21. *$P < 0.05$. For two-group comparisons, we used Hedges's g with 95% confidence intervals ($n<20$). Hedges's g values for C and D were 4.219 and 1.060, respectively.

(E-F) Absolute counts of CAR$^+$CD8$^+$ (E) and CAR$^+$CD4$^+$ (F) T cells per spleen on day 21. P values were determined by unpaired Student's t-test. *$P < 0.05$; n.s., not significant. For two-group comparisons, we used Hedges's g with 95% confidence intervals ($n<20$). Hedges's g values for E was 2.691.

Figure S7

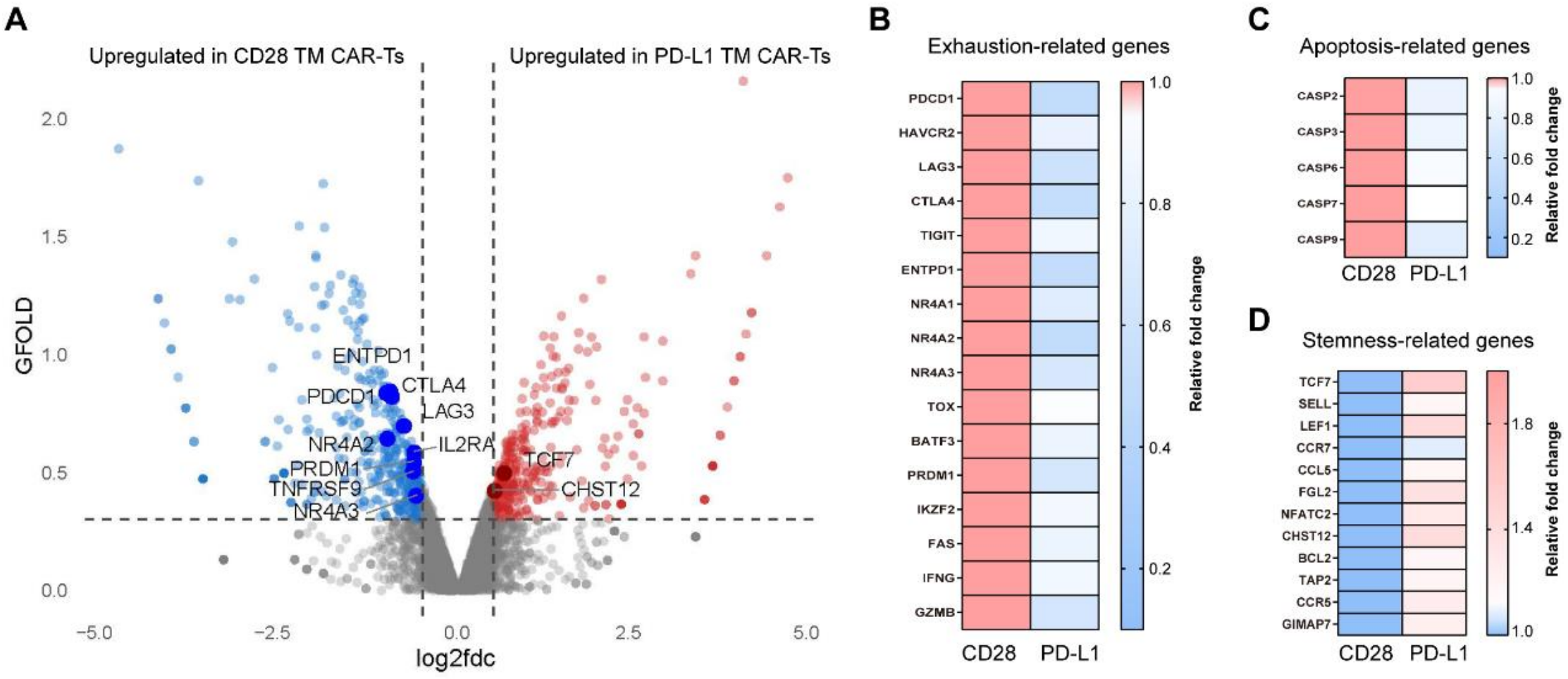

Figure S7. Transcriptomic validation of PD-L1 TM benefits in GD2 CAR-T cells (related to Figure 5).

(A) Volcano plot of differentially expressed genes between CD28 TM and PD-L1 TM GD2 CAR-T cells.

(B) mRNA levels of exhaustion-related genes. Data represent the fold change of PD-L1 TM relative to CD28 TM.

(C) mRNA levels of apoptosis-related genes. Data represent the fold change of PD-L1 TM relative to CD28 TM.

(D) mRNA levels of memory- and stemness-related genes. Data represent the fold change of PD-L1 TM relative to CD28 TM.
